# RNA‐binding protein RBM24 represses colorectal tumourigenesis by stabilising PTEN mRNA

**DOI:** 10.1002/ctm2.383

**Published:** 2021-10-12

**Authors:** Rong Mu Xia, Tao Liu, Wen Gang Li, Xiu Qin Xu

**Affiliations:** ^1^ Institute of Stem Cell and Regenerative Medicine School of Medicine Xiamen University Xiamen Fujian People's Republic of China; ^2^ Department of Hepatobiliary Surgery School of Medicine Xiang'an Hospital of Xiamen University Xiamen University Xiamen Fujian People's Republic of China

**Keywords:** colorectal cancer, mRNA stability, PTEN, RBM24, tumourigenesis

## Abstract

**Background:**

RNA‐binding motif protein 24 (RBM24) functions as a splicing regulator, which is critical for organ development and is dysregulated in human cancers. Here, we aim to uncover the biological function of RBM24 in colorectal tumourigenesis.

**Methods:**

Xenograft tumour model, Rbm24 knockout and Apc^min/+^ mouse models were utilised. Colorectal cancer cells overexpressing or silencing RBM24 were established. RNA immunoprecipitation (RIP) assay was conducted to detect protein‐RNA associations. Gene expression was measured by immunohistochemistry, western blotting, or quantitative PCR (qPCR).

**Results:**

Rbm24‐knockout mice developed spontaneous colorectal adenomas with lower expression of phosphatase and tensin homolog (PTEN). Immunohistochemical staining for the proliferation markers Ki‐67 and pHH3 and BrdU assay showed intestinal hyperplasia in Rbm24‐knockout mice compared to wild‐type mice. RBM24 expression in colorectal adenoma tissues of Apc^min/+^ mouse was downregulated compared with adjacent normal samples and was positively correlated with PTEN expression. In vitro, RBM24 overexpression suppressed cell proliferation, migration, invasion and increased sensitivity to 5‐FU or cisplatin in CRC cells. Mechanistically, RBM24 maintained PTEN mRNA stability by directly binding to the GT‐rich region at positions 8101–8251 in the 3′‐UTR of PTEN mRNA, prolonging the half‐life of PTEN mRNA, thereby increasing PTEN expression. Hence, low expression of RBM24 downregulated PTEN mRNA, causing the activation of PI3K‐Akt signalling in CRC cells. Furthermore, RBM24 expression in CRC tissues was lower than adjacent normal samples. RBM24 expression was positively correlated with PTEN expression and negatively correlated with Ki‐67 level. CRC patients with high RBM24 expression had a favourable outcome.

**Conclusions:**

Taken together, RBM24 expression is markedly lower in colorectal tumours than in para‐carcinoma tissues. Rbm24‐knockout mice develop spontaneous colorectal adenomas. RBM24 directly binds and stabilises PTEN mRNA, which could cause the suppression of CRC cell proliferation, migration and invasion, thereby repressing colorectal tumourigenesis. These findings support the tumour‐suppressive role of RBM24. Targeting RBM24 holds strong promise for the diagnosis and treatment of CRC.

## Graphical abstract

1. RBM24 knockout mice develop spontaneous colorectal adenomas with a lower level of PTEN in comparison with wild‐type mice.
2. RBM24 maintains PTEN mRNA stability by directly binding to the GT‐rich region at positions 8101–8251 in the 3′‐UTR of PTEN mRNA.
3. Decreased PTEN expression mediated by RBM24 contributes to the activation of the PI3K/Akt signalling pathway, promoting colorectal cancer (CRC) development.

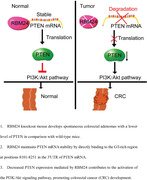

## 1 BACKGROUND

Colorectal cancer (CRC) causes approximately 900 000 deaths globally each year.^1^ Mounting evidence suggests that genetic mutations, epigenetic modifications, chronic inflammation, diet, lifestyle and intestinal flora are the main risk factors for CRC.^2^, ^3^ CRC patients diagnosed at an early stage have a 90% probability of being cured by surgical resection.^4^ However, due to insidious onset and unrecognised clinical symptoms, most patients with CRC are diagnosed when they are already in the advanced stage, resulting in poor treatment outcomes.^4^, ^5^ Although the pathogenesis of CRC has been extensively studied, the underlying mechanisms of CRC initiation and progression have not been fully elucidated.

Tumour suppressor gene mutation or inactivation contributes to colorectal carcinogenesis,^6^, ^7^ for example, mutations in APC,^8^ P53,^9^ and PTEN.^10^ These mutations show significant association with the development of CRC. Although previous studies have shown that P53 is mutated in approximately half of human tumours and PTEN mutation occurs in more than 20%, a substantial proportion of individuals without these mutations will also develop CRC.^11^, ^12^ Hence, a better understanding of the precise pathogenic mechanisms involved in colorectal tumourigenesis is warranted for the prevention, diagnosis and treatment of CRC.

RNA‐binding protein (RBP) contains mRNA‐binding domain(s), which can directly bind to intracellular mRNA and further regulate mRNA maturation, transport, localisation and translation.^13^, ^14^ RBPs play important role in individual development, homeostasis, pathological or physiological processes of cancer and other diseases.^15^, ^16^, ^17^ Via specific RNA‐binding domains, RBPs can recognise specific RNA sequences.^16^ RBM24, a member of the RBP family, has a highly conserved RNA‐recognition motif (RRM) and four exons that encode a 236 amino‐acids protein (24.7 kDa). RBM24 gene is located on human chromosome 6 and mouse chromosome 13. RBM24 has been reported to be significantly upregulated during myocardial differentiation of embryonic stem cells.^18^, ^19^ Previous studies have demonstrated that RBM24 regulates alternative splicing switches^18^, ^20^ and also directly binds to the GU(A)GUGU site on RNA, thereby affecting mRNA maturation or translation.^20^ RBM24 regulates the mRNA stability of P53, P21 and P63, which in turn influence protein translation.^21^, ^22^, ^23^, ^24^ Recently, it has been revealed that RBM24 has an effect on eye development by adjusting the poly(A) tail length and translation efficiency in the lens.^25^ These studies indicate that RBM24 exhibits extensive regulatory functions. Therefore, its biological activity in other organs deserves further investigation.

During the construction process of our Rbm24 knockout mouse model, we unexpectedly detected a certain number of adenomas within the intestine of tamoxifen‐induced Rbm24‐knockout mice. Herein, we performed experiments both in vitro and in vivo to understand the mechanisms underlying the involvement of RBM24 in colorectal tumourigenesis. Our findings showed that Rbm24 knockout promoted colorectal tumourigenesis. RBM24 expression in human colorectal specimens and mouse colorectal adenoma tissues was significantly lower than in controls. RBM24 stabilised PTEN, a tumour suppressor gene, mRNA and controlled the proliferative and metastatic capabilities of CRC cells. RBM24 exerts a tumour suppressive role in CRC and targeting RBM24 holds strong promise for CRC diagnosis and treatment.

## 2 METHODS

### 2.1 Patients and colorectal tissues

This study was approved by the Ethics Committee of Xiamen University and written informed consent was obtained from all patients (Approval no. XDYX2021008). A total of 36 CRC samples and paired adjacent normal colorectal tissues samples were collected. All patients enrolled in this study haven't received drug treatment, chemo‐ or radiotherapy prior to surgical resection. Samples were prepared for western blotting, qPCR and immunohistochemistry (IHC) assays. The patient details are listed in Table 1.

**TABLE 1:** The clinicopathological factors of CRC patients (36 cases)

Characteristics	Number of cases (%)
Age (year)	
≤60	17 (47.2)
>60	19 (52.8)
Gender	
Male	21 (58.3)
Female	15 (41.7)
Depth of tumour invasion	
T1‐2	19 (52.8)
T3–4	17 (47.2)
TNM stage	
I+II	26 (72.2)
III	10 (27.8)
Lymph node metastasis	
N0	20 (55.6)
N1+N2	16 (44.4)
Distant metastasis	
M0	19 (52.8)
M1+M2	17 (47.2)

### 2.2 Animal experiments

Apc^min/+^ mice were obtained from Shanghai Model Organisms Center (*N* = 18). C57BL/6 mice were used as the wild‐type (WT) group (*N* = 18). UBC‐Cre/ERT2 mice were generously donated by Professor Chen Yongxiong (Ophthalmology Center, School of Medicine, Xiamen University). Rbm24^loxp/loxp^ mice were generated by inserting two LoxP sites on the flank of second and third exons of the *Rbm24* gene (Gene ID: 666794).^20^ Then, Rbm24^loxp/loxp^ mice were bred with UBC‐Cre/ERT2 mice to generate CreERT2‐Rbm24^loxp/loxp^ mice.^26^ To generate Rbm24‐knockout mice, CreERT2‐Rbm24^loxp/loxp^ mice were treated with tamoxifen (100 mg/kg/d, ip) for 5 consecutive days (2‐month‐old; *N* = 32). C57BL/6 mice given the same dose of tamoxifen were used as the WT control group (*N* = 32). Knockout efficiency was validated by qPCR and Western blotting analysis.

For xenograft tumour models, female BALB/c nude mice (6‐week‐old) were obtained from Shanghai Model Organisms Center. The nude mice (*N* = 6 mice per group) were injected subcutaneously with 2 × 10^6^ shRBM24‐HCT116 cells or 2 × 10^6^ OERBM24‐HCT116 cells or 2 × 10^6^ control cells (suspended in 100 μl of sterile PBS) at the right flanks. The long (*L*) and short diameter (*W*) of tumours were detected with callipers every week and analysed by the formula: volume = 0.5 × *L* × *W* ^2^. Mice were killed on day 42 under isoflurane anaesthesia. Next, the transplanted tumours were stripped and photographed. For lung metastasis model, 1 × 10^5^ shRBM24‐HCT116 cells or 1 × 10^5^ OERBM24‐HCT116 cells or 1 × 10^5^ control cells (diluted in 50 μl of sterile PBS) were injected via tail vein injection. Metastatic lung nodules were observed after 6 weeks.

All mice were kept in specific pathogen‐free (SPF) conditions with 12‐h light/12‐h dark cycle and free access to food and water. All the experimental procedures were operated in accordance with the guidelines and regulations of animal experimentation approved by the Experimental Animal Center of Xiamen University. The Experimental Animal Committee of Xiamen University approved all animal procedures (Approval ID: SCXK2013‐0006).

### 2.3 Cell culture and construction of cell line silencing or overexpressing RBM24

Human HCT116, SW480 and LoVo cell lines were purchased from the Cell Bank of the Chinese Academy of Sciences (Shanghai, China). CRC cells were cultivated with DMEM medium supplemented 10% FBS (Gibco) and 1% penicillin‐streptomycin (Gibco) at 37°C under 5% CO_2_ in saturated humidity conditions.

To establish RBM24‐silenced cell lines, a specific short hairpin RNA (shRNA) of RBM24 (Table 3) was designed and packaged into lentiviral particles. After 48 h of infection, the cells were subjected to 1 μg/ml of puromycin for more than 72 h. RBM24 expression was examined using qPCR and Western blotting analysis.

To construct RBM24‐overexpressing cell lines, lentiviruses overexpressing RBM24 were established. Next, the cells were infected with the lentiviruses and stable cell populations were selected for puromycin as described above. The efficiency of RBM24 overexpression was measured using qPCR analysis and western blotting assays.

### 2.4 Plasmid and small interfering RNA (siRNA) transfections

*pcDNA3.1*‐PTEN plasmid or the control vector (Promega) were transfected into CRC cells using Lipofectamine 3000 (ThermoFisher Scientific) following instructions, respectively. For siRNA transfections, CRC Cells were transfected with 1.5 μg/ml of siRNA using Lipofectamine RNAiMAX (Invitrogen).

### 2.5 Preparation of tissue sections of mouse intestine

Mice were sacrificed by cervical dislocation. Mouse colon and parts of the small intestine were excised and washed using pre‐cooled PBS to ensure that the contents of the intestine were rinsed clean. Afterwards, the intestine was flattened and photographed. Thereafter, mouse intestinal tissues were fixed in 4% buffered paraformaldehyde for 48 h; rinsed 3 times with PBS for 5 min each time; dehydrated with 10% sucrose solution, followed by gradient dehydration using 20% and 30% sucrose solution in sequence. Next, the OCT‐embedded tissues were snap‐frozen to –80℃. Five‐micrometre‐thick sections were prepared for immunofluorescence.

### 2.6 Immunofluorescence

Antigen retrieval was performed using 0.01 M citrate buffer (pH 6.0) at 95°C for 20 min. The sections were processed for 0.25% Triton X‐100 treatment for 15 min, blocked with 5% goat serum in PBS for 1 h at room temperature (RT) and then incubated with corresponding primary antibodies (prepared in goat serum): anit‐RBM24 (Abcam, Cat no: ab94567, 1:300), anti‐Ki‐67 (Abcam, Cat no: ab15580, 1:300), anti‐pHH3 (Abcam, Cat no: ab5176, 1:300), anti‐PTEN (Santa Cruz, Cat no: SC‐7974, 1:300) overnight at 4°C. After washing five times with PBS, the sections were incubated with the appropriate secondary antibodies (prepared with goat serum) AlexaFluor^®^488‐conjugated goat anti‐rabbit IgG (H+L) (Life Technologies, Cat no: A‐11008, 1:500), AlexaFluor^®^488‐conjugated goat anti‐mouse IgG (H+L) (Life Technologies, Cat no: A‐11001, 1:500), or AlexaFluor^®^555‐conjugated goat anti‐rabbit IgG (H+L) (Life Technologies, Cat no: A‐21428, 1:500) at RT in the dark for 1 h following washes with PBS. Next, slides were treated with the fluorescent blue dye Hoechst 33258 (Sigma, 1:1000) at RT for 10 min. After washing with PBS again, the slides were sealed with 50% glycerin. Representative pictures were photographed using a fluorescence microscope (Olympus, Japan).

### 2.7 Immunochemistry

Paraffin‐embedded tissue sections were treated with H_2_O_2_ solution at RT for 10 min. Following washing with PBS, antigen retrieval was conducted as described above. Next, the sections were blocked with 5% goat serum in PBS at RT for 1 h and then incubated with primary antibodies (anti‐RBM24, Abcam, Cat no: ab94567, 1:300; anti‐PTEN, Santa Cruz, Cat no: SC‐7974, 1:300; or anti‐Ki‐67, Abcam, Cat no: ab15580, 1:300) overnight at 4°C. Subsequently, the sections were subjected to HRP‐conjugated secondary antibody for 1 h at RT following washing with PBS. Color was developed using DAB solution at RT for 5 min. Tissue sections were counterstained with haematoxylin staining for 30 s, followed by dehydration in differential alcohol gradients. Sections were coverslipped with neutral balsam and then observed under microscope (Olympus, Japan).

### 2.8 Western blotting

Tissues or cells were treated with lysis buffer containing 1% PMSF. Protein quantitation was determined using the bicinchoninic acid assay. Thirty micrograms of protein was run on 10% SDS‐PAGE gel. Subsequently, the gel‐separated proteins were transferred onto PVDF membranes by wet transfer. The membranes were blocked with 5% non‐fat milk for 2 h at RT and then incubated overnight with the primary antibody at 4°C, followed by incubation with goat anti‐mouse (Boster Biological Technology Co. Ltd, Cat no: BA1075, 1:2000) or goat anti‐rabbit (Boster Biological Technology Co. Ltd, Cat no: BA1054, 1:2000) IgG‐HRP secondary antibody, for 1 h at RT. Immunoblots were visualised using a chemiluminescence kit (Millipore). β‐actin or GAPDH was selected as the internal control for gene expression normalisation based on the molecular weight of protein. The primary antibodies and corresponding secondary antibodies used in present study and their appropriate working dilutions are listed in Table 2.

**TABLE 2:** Primary antibodies and corresponding secondary antibodies used in this study and their optimal working dilutions

Antibodies	Manufacturer	Catalogue No.	Working dilutions
GAPDH	Abcam	ab8227	1:5000
β‐actin	Cell Signaling Technology	4970	1:4500
RBM24	Abcam	ab94567	1:1000
PTEN	Santa Cruz	sc‐7974	1:2500
P21	Santa Cruz	sc‐6246	1:3000
MMP‐2	Cell Signaling Technology	40994	1:3500
MMP‐9	Cell Signaling Technology	13667	1:3500
E‐Cadherin	Abcam	ab227639	1:4000
Vimentin	Cell Signaling Technology	5741	1:3000
pHH3	Abcam	ab14955	1:1000
CyclinD1	Abcam	ab226977	1:4500
CDK4	Abcam	ab137675	1:4500
Ki‐67	Abcam	ab15580	1:500
Phosphorylated H2AX (γH2AX)	Cell Signaling Technology	9718	1:2500
H2AX	Cell Signaling Technology	7631	1:3500
ATM	Cell Signaling Technology	2873	1:3500
Phosphorylated ATM (p‐ATM)	Cell Signaling Technology	13050	1:1500
ATR	Cell Signaling Technology	13934	1:3500
Phosphorylated ATR (p‐ATR)	Cell Signaling Technology	2853	1:1500
PI3K	Abcam	ab32089	1:3500
Akt	Cell Signaling Technology	9272	1:3500
Phosphorylated Akt (p‐Akt)	Abcam	ab131443	1:1500
Cleaved‐caspase3	Cell Signaling Technology	9664	1:1500
Cleaved‐PARP	Cell Signaling Technology	5625	1:1500

### 2.9 Bromodeoxyuridine (BrdU) staining

BrdU (Sigma, B5002‐1G, 10 mg/ml) was dissolved in PBS. Twelve hours before excising the tissues, BrdU (50 mg/kg) solution was injected intraperitoneally into mice. The sections were prepared as mentioned above and treated with 1 nmol of hydrochloric acid at 37°C for 45 min. Following washing with 0.1 mol sodium borate (pH 8.4) and blocking with 5% goat serum in PBS at RT for 2 h, sections were sequentially incubated overnight with anti‐BrdU antibody (Abcam, Cat no: ab6326, at a dilution 1:300) at 4°C and AlexaFluor^®^488 conjugated goat anti‐rat IgG (H+L) secondary antibody (Life Technologies, Cat no: A‐11006, with a dilution 1:500) at RT for 1 h. Nuclei were stained by Hoechst dye. Representative pictures were taken with fluorescence microscope (Olympus, Japan).

### 2.10 Quantitative polymerase chain reaction (qPCR)

Total RNA was prepared using TRIzol reagent (Life Technology) following the guidance. EasyScript^®^ First‐Strand cDNA Synthesis SuperMix (Beijing TransGen Biotech Co., Ltd, China) and TransScript^®^ One‐Step RT‐PCR SuperMix (Beijing TransGen Biotech Co., Ltd, China) were used to synthesise cDNA and for qPCR analysis following the manufacturer's instructions, respectively. Furthermore, the target gene amplification was confirmed using agarose gel electrophoresis. All primers are listed in Table 3. Gene expression was normalised to the expression level of housekeeping gene GAPDH.

**TABLE 3:** The sequences of all primers used in this study

Gene	Forward	Reverse
*hGAPDH*	5′‐GCAAAGTGGAGATTGTTGCCAT‐3′	5′‐ CCTTGACTGTGCCGTTGAATTT ‐3′
*mGAPDH*	5′‐AGGTCGGTGTGAACGGATTTG‐3′	5′‐TGTAGACCATGTAGTTGAGGTCA‐3′
*hRBM24*	5′‐CCAAGGATCATGCAACCAG‐3′	5′‐GCAGGTATCCCGAAAGGTCT ‐3′
*mRBM24*	5′‐GGGGCTACGGATTTGTCACC‐3′	5′‐TGGCTGCATGATTCTTGGTTT‐3′
*hPTEN*	5′‐TGGATTCGACTTAGACTTGACCT‐3′	5′‐GGTGGGTTATGGTCTTCAAAAGG‐3′
*mPTEN*	5′‐TGGATTCGACTTAGACTTGACCT‐3′	5′‐GCGGTGTCATAATGTCTCTCAG‐3′
*hP21*	5′‐AATTGGAGTCAGGCGCAGAT‐3′	5′‐CGAAGAGACAACGGCACACT‐3′
*mP21*	5′‐CCTGGTGATGTCCGACCTG‐3′	5′‐CCATGAGCGCATCGCAATC‐3′
*hMMP‐2*	5′‐TACAGGATCATTGGCTACACACC‐3′	5′‐GGTCACATCGCTCCAGACT‐3′
*mMMP‐2*	5′‐CAAGTTCCCCGGCGATGTC‐3′	5′‐TTCTGGTCAAGGTCACCTGTC‐3′
*hMMP‐9*	5′‐AATCTCACCGACAGGCAGCT‐3′	5′‐CCAAACTGGATGACGATGTC‐3′
*mMMP‐9*	5′‐ CTGGACAGCCAGACACTAAAG‐3′	5′‐CTCGCGGCAAGTCTTCAGAG‐3′
*hE‐Cadherin*	5′‐CGAGAGCTACACGTTCACGG‐3′	5′‐GGGTGTCGAGGGAAAAATAGG‐3′
*hVimentin*	5′‐GCCCTAGACGAACTGGGTC‐3′	5′‐GGCTGCAACTGCCTAATGAG‐3′
*hCyclinD1*	5′‐TGGAGCCCGTGAAAAAGAGC‐3′	5′‐TCTCCTTCATCTTAGAGGCCAC‐3′
*hCDK4*	5′‐TTCGTGAGGTGGCTTTACTG‐3′	5′‐GATATGTCCTTAGGTCCTGGTCT‐3′
*CRE*	5′‐ATTTGCCTGCATTACCGGTC‐3′	5′‐ATCAACGTTTTCTTTTCGG‐3′
*mRBM24‐FRT‐TF2*	5′‐CATGGATGTTGGTGGTGCTGTC‐3′	5′‐GACCTGGCGGTAGACAGACATTG‐3′
*ShRNA hRBM24*	5′‐GCG AGC AAT ATG TAG CTT GAA‐3′	
*si PTEN*	5′‐TCTTCAAAAGGATATTGTGCA‐3′	

### 2.11 Haematoxylin and eosin (HE) staining

Tissue sections, as described above, were stained with haematoxylin at 60°C for 60 s, subjected to 1% hydrochloric acid ethanol treatment for 3 s, rinsed in running tap water, stained with eosin for 60 s, rinsed with distilled water for 2 s and further dehydrated with ethanol and xylene in order. The slides were sealed with neutral balsam. Images were photographed under an optical microscope (Olympus, Japan).

### 2.12 Cell Counting Kit 8 (CCK‐8) assay

CRC cells overexpressing or silencing RBM24 (1 × 10^3^ cells/well) were seeded into 96‐well dishes and cultured overnight. CCK‐8 regent was added into cells at each time point, followed by incubation for 2 h. Cell viability was assessed by detecting the absorbance at 450 nm using a Microplate Reader (Bio‐Rad). Cell‐free medium with CCK‐8 reagent was included as the negative control.

### 2.13 Colony‐formation assay

To assess the role of RBM24 in CRC cells, 6‐well plates were seeded with 1 × 10^3^ cells and cultured for 10 days. The culture medium was changed every 2 days. Subsequently, the supernatant was discarded to remove the floating cells. After fixing with 75% ethanol at RT for 10 min, the cells were stained with 1% crystal violet (CV) for 30 min. Ten fields were reviewed in each well and were randomly selected under a microscope (Olympus, Japan). The number of colonies (≥50 cells) and the average number of cells per clone were calculated.

### 2.14 Transwell migration/invasion (with Matrigel) assays

Briefly, 5 × 10^5^ cells per well were plated into the upper chamber. For transwell invasion assay, the upper chamber was pre‐coated with 5 mg/ml of Matrigel. The lower transwell chamber contained 500 μl of culture medium. Twelve hours later, the cells were stained using 1% crystal violet as described above. The migrated or invaded cells were counted (five fields of view for each group).

### 2.15 Cell apoptosis and cell cycle

Cell apoptosis and cell cycle were measured by flow cytometric analysis. Cell apoptosis was tested by Annexin V‐FITC and PI double‐staining (Beijing Solarbio Science and Technology Co., LTD., China). Cell cycle was evaluated by PI staining as suggested by manufacturer's protocol. Data were analysed using Flowjo 7.6 software. All experiments were independently performed a minimum of three times.

### 2.16 Wound‐healing assay

When the cells in each group reached 95% confluence, the confluent cell monolayer was scratched with a sterile pipette tip (0 h). Afterwards, the wound closure at different time points was monitored under the microscope (Olympus, Japan).

### 2.17 RNA immunoprecipitation assay (RIP)

Cells transfected with RBM24‐Flag plasmids were harvested 48 h post‐transfection and then fully lysed using RIP lysis buffer. Anti‐Flag beads (50 μl) were pre‐washed five times with NT2 buffer, then 50 μl of magnetic beads were added into 800 μl of cell lysate, following incubation overnight at 4°C with shaking. Magnetic beads were precipitated by magnetic separation and quickly washed twice with NT2 buffer. The immunoprecipitated pellet was collected and prepared for western blotting and qPCR analysis. Magnetic beads coated with anti‐IgG antibodies were used as negative controls.

### 2.18 Statistical analysis

Data, from a minimum of at least three independent experiments, were analysed using SPSS (version 21.0, IBM Corp.) and expressed as the mean ± standard deviation (SD). Two‐tailed Student's *t*‐test, one‐way analysis of variance (ANOVA), post hoc least significant difference (LSD) test, Cox test, or Pearson's correlation test was applied for data analysis. GraphPad Prism 5.0 software (GraphPad Software) was used for graphing. Asterisk represented a *p* value less than .05.

## 3 RESULTS

### 3.1 Rbm24‐knockout mice develop spontaneous colorectal adenomas

**FIGURE 1:**
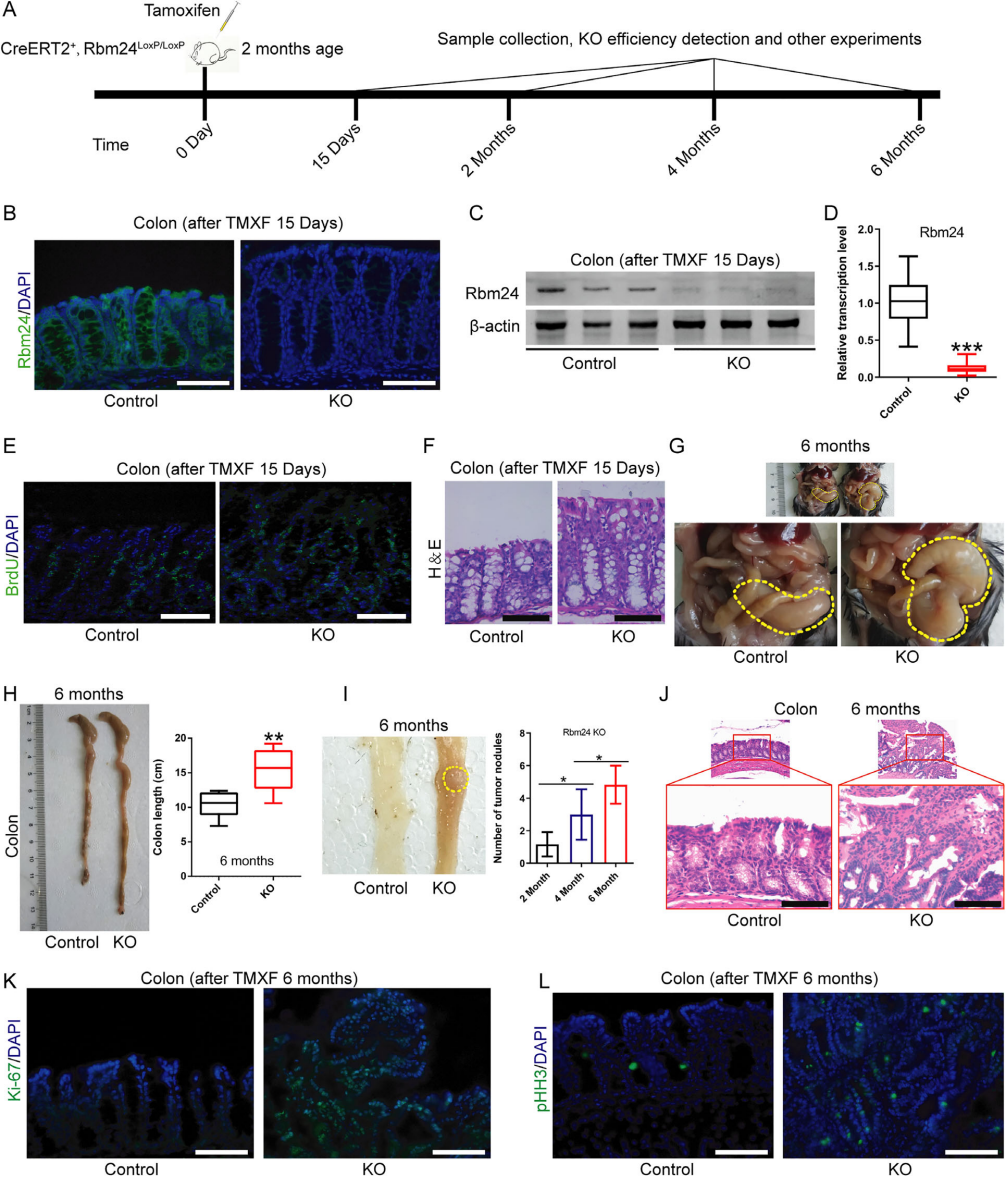
Rbm24‐knockout mice develop spontaneous colorectal adenomas. (A) Schematic diagram of animal experiments (*N* = 32 mice per group). (B, C, D) Rbm24 knockout efficiency was detected by (B) immunofluorescence, (C) Western blotting and (D) qPCR analysis after daily intraperitoneal injection of tamoxifen (100 mg/kg) for 15 days. (E, F) In CreERT2/Rbm24^loxp/loxp^ mice 15 days after tamoxifen administration, BrdU‐labelled cells were detected by (E) immunofluorescence and (F) colonic epithelial hyperplasia was assessed by HE staining. (G) Representative images of the colon of Rbm24‐knockout mice 6 months after tamoxifen injection. (H) Representative images of the colon and quantification of colonic length in Rbm24‐knockout mice 6 months after tamoxifen injection. (I) Tumour nodules and quantification in Rbm24‐knockout mice 2 months (*N* = 7 mice per group), 4 months (N = 8 mice per group), 6 months (N = 17 mice per group) after tamoxifen injection, respectively. (J) HE staining of the colonic tumour nodules in Rbm24‐knockout mice; (K) Ki‐67 and (L) pHH3 expression in colonic tumour and adjacent normal tissues were evaluated by immunofluorescence. Data were expressed as the mean ± SD from at least three independent experiments and analysed using two‐tailed Student's *t*‐test or one‐way analysis of variance (ANOVA) with post hoc Fisher's Least Significant Difference (LSD) test. *p<.05, **p<.01, ***p<.001. KO, knockout. Scale bars = 50 μm

Previously, Yang et al. confirmed that systemic Rbm24 knockout resulted in embryonic lethality.^27^ Hence, Rbm24‐inducible knockout mouse model was constructed (Figure S1A–D).^28^ Tamoxifen (100 mg/kg daily for 5 days) was injected intraperitoneally into 8‐week‐old CreERT2‐Rbm24^loxp/loxp^ mice (*N* = 32) to generate Rbm24 conditional knockout mice. Wild type (WT) mice were administrated with the same dose of tamoxifen (Figure 1A). Knockout efficiency was validated using immunofluorescence, western blotting and qPCR analysis (Figure 1B–D). We found that BrdU‐positive cells (Figure 1E) in the colon and the expression levels of Ki‐67 and pHH3 protein in the intestine (Figure S1E–H) of Rbm24‐knockout mice increased significantly compared with the WT mice 15 days after tamoxifen administration. Besides, colonic wall thickening in the Rbm24‐knockout mice was observed by HE staining (Figure 1F). At 6 months after Rbm24 knockout, an enlargement of the colon in mice was observed (Figure 1G); the colorectal segment was strikingly longer than that of WT mice (Figure 1H) and the number of adenomas increased significantly (Figure 1I, J). Immunofluorescence also confirmed a significant increase in Ki‐67‐positive (Figure 1K) and pHH3‐positive cells (Figure 1L) in adenoma tissues. No adenoma was found in WT mice at 6 months. These data suggest that Rbm24 knockout promotes the development of colorectal adenomas in mice.

### 3.2 RBM24 overexpression suppresses malignant behaviours in CRC cells

**FIGURE 2:**
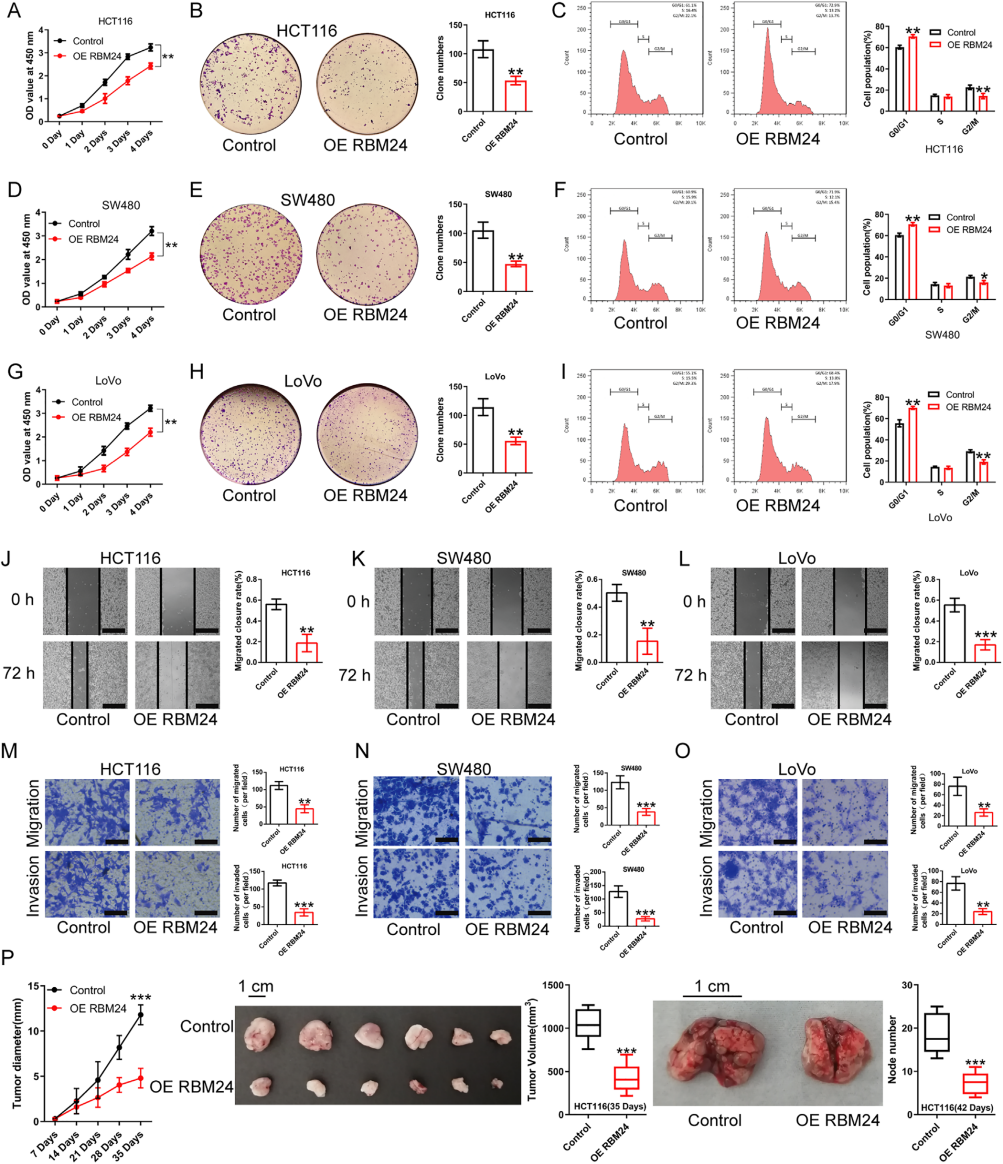
RBM24 overexpression represses the proliferation, migration and invasion of CRC cells. (A‐I) Proliferation and cell cycle of RBM24‐overexpressing (A, B, C) HCT116, (D, E, F) SW480 or (G, H, I) LoVo cells were measured using CCK‐8, colony formation assays and flow cytometry analysis. (J–O) Overexpression of RBM24 restrained the migration and invasion of HCT116, SW480 and LoVo cells; the migratory ability of CRC cells was evaluated by wound‐healing assay and transwell migration assay; the invasive ability of CRC cells was assessed by transwell invasion assay. Scale bar (J, K and L) = 100 μm. Scale bar (M, N and O) = 50 μm. (P) RBM24 overexpression in HCT116 cells suppressed CRC cell growth and lung metastasis in nude mice (*N* = 6 mice per group). Data were analysed by two‐tailed unpaired Student's *t*‐test. Error bars represented as SD from at least three independent experiments. *p<.05, **p<.01, ***p<.001

**FIGURE 3:**
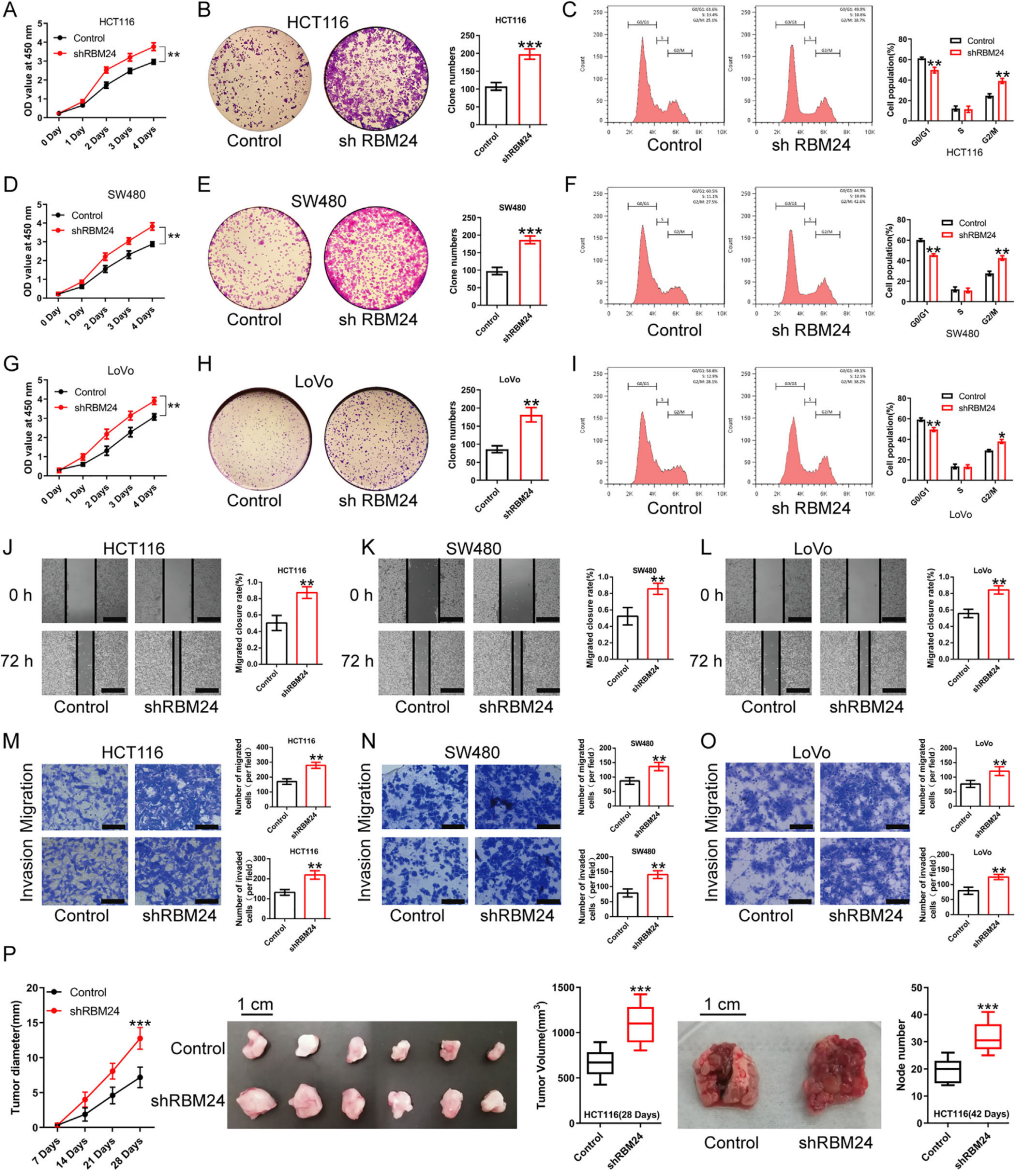
RBM24 knockdown promotes the proliferation, migration and invasion of CRC cells. (A–I) CCK‐8, colony formation assay and cell cycle analysis showed that RBM24 knockdown facilitated cell proliferation, enhanced the colony formation capability and promoted cell cycle progression of (A, B, C) HCT116, (D, E, F) SW480 and (G, H, I) LoVo cells. (J–O) RBM24 knockdown facilitated the migration and invasion of (J, M) HCT116, (K, N) SW480 and (L, O) LoVo cells; the migratory ability of CRC cells was evaluated by wound‐healing assay and transwell migration assay; the invasive capability of CRC cells was monitored by transwell invasion assay. Scale bar (J, K and L) = 100 μm. Scale bar (M, N and O) = 50 μm. (P) RBM24 knockdown in HCT116 cells promoted CRC cell growth and lung metastasis in nude mice (*n* = 6 mice per group). Data were analysed by two‐tailed unpaired Student's *t*‐test. Error bars represented as SD from at least three independent experiments. *p<.05, **p<.01, ***p<.001

To understand the role of RBM24 in CRC, we performed cell proliferation assay, CCK‐8 assay, cell cycle analysis and colony formation assay. We found that RBM24 overexpression repressed cell proliferation, contributed to cell cycle arrest at the G0/G1 phase and reduced colony‐forming capability of human CRC cells (HCT116, SW480 and LoVo) (Figure 2A–I). To further investigate the two typical characteristics (invasion and metastasis) of cancer cells,^29^, ^30^ we performed wound‐healing and transwell assays. We found enforced expression of RBM24 suppressed migration and invasion of CRC cells as compared to the matched controls (Figure 2J–O). In addition, RBM24 overexpression restrained tumour growth and lung metastasis in vivo (Figure 2P). Conversely, RBM24 knockdown yielded opposing results (Figure 3A–P). These results indicate that RBM24 suppresses CRC cell growth, both in vitro and in vivo.

### 3.3 RBM24 regulates PTEN expression and downstream signalling of PTEN

**FIGURE 4:**
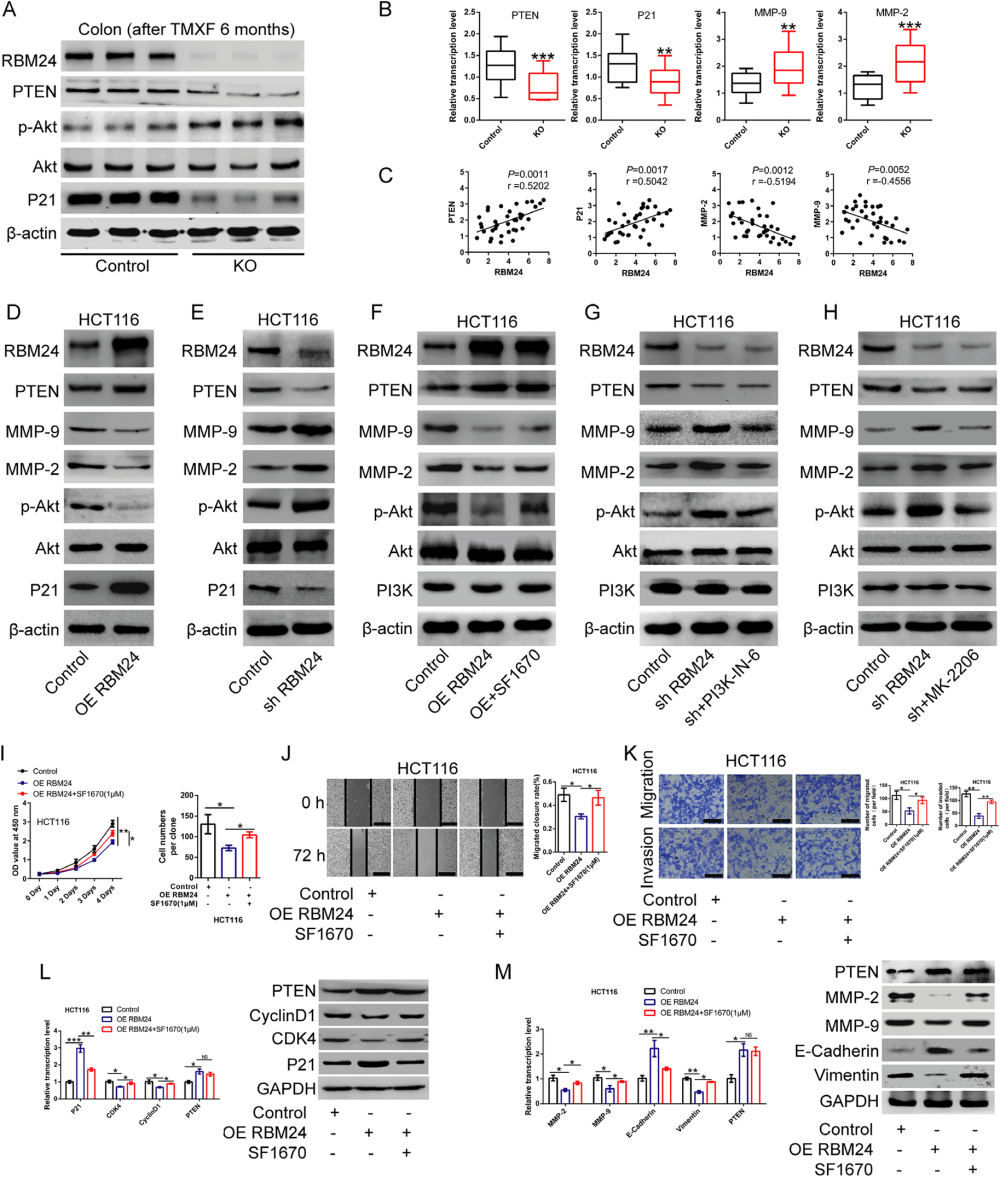
Effect of RBM24 overexpression or knockdown on gene expressions. (A) The protein levles of PTEN, Akt, p‐Akt, P21 and Rbm24 in colon tissues of Rbm24‐knockout mice and wild type mice 6 months post‐injection was detected by Western blotting analysis. (B) The mRNA expression of PTEN, P21, MMP‐2, MMP‐9 and (C) their correlation with Rbm24 expression in colonic tissues of Rbm24‐knockout and wild type mice. (D, E) The protein levels of RBM24, PTEN, P21, Akt, MMP‐2, MMP‐9 and the phospho‐Akt in HCT116 CRC cells (D) overexpressing or (E) silencing RBM24 were analyzed by Western blotting assay. (F–H) Protein levels of RBM24, PTEN, PI3K, Akt, MMP‐2, MMP‐9 and the phospho‐Akt (F) in RBM24‐overexpressing HCT116 cells following treatment with 1 μM of PTEN inhibitor SF1670 for 48 h or (G, H) in RBM24‐knockdown HCT116 cells following treatment with PI3K inhibitor 10 nM of PI3K‐IN‐6 or 20 nM of Akt inhibitor MK‐2206 for 48 h were measured by Western blotting analysis. (I) Overexpression of RBM24 repressed the proliferation and colony formation of HCT116 cells, while SF1670 treatment (1 μM) reversed the results caused by RBM24 overexpression. (J, K) After overexpression of RBM24 following treatment with 1 μM of SF1670 for 48 h, the migration and invasion of HCT116 cells were tested by (J) wound‐healing assay and (K) transwell invasion assay. J, scale bar = 100 μm. K, scale bar = 50 μm. (L) After overexpression of RBM24 and treatment with 1 μM of SF1670 for 48 h, the expression levels of P21, CDK4, CyclinD1 and PTEN in HCT116 cells were evaluated by qPCR (left) and Western blotting analysis (right). (M) After overexpression of RBM24 and treatment with 1 μM of SF1670 for 48 h, the expression levels of MMP‐2, MMP‐9, E‐Cadherin, Vimentin and PTEN were detected using qPCR (left) and Western blotting analysis (right). Data were expressed as the mean ± SD from at least three independent experiments and analysed using one‐way ANOVA with post hoc LSD test. *p<.05, **p<.01, ***p<.001

In our preliminary experiments, we observed an association between the expression of RBM24 and PTEN. PTEN, an important negative regulator of the PI3K pathway, is a classical tumour suppressor in various type of cancers. We assumed that RBM24 may affect PTEN expression and its downstream PI3K/Akt signalling pathway. Western blotting and qPCR analysis revealed that PTEN expression was significantly decreased and positively correlated with RBM24 expression in tumour tissues of RBM24‐knockout mice. Akt is a key molecule of PI3K/Akt signalling. In present study, Akt phosphorylation was significantly upregulated in tumour tissues of RBM24‐knockout mice, while there was no difference in the expression of total Akt in tumour tissues of RBM24‐knockout mice compared with WT mice. Similarly, the expression of Matrix metalloproteinases 2 and 9 (MMP2 and MMP9), downstream molecules of the PI3K/Akt pathway, was markedly increased and correlated inversely with RBM24 expression, whereas P21 expression showed a significant positive correlation with RBM24 expression in tumour tissues of RBM24‐knockout mice in comparison to WT mice (Figure 4A–C).

Consistent with the above in vivo findings, RBM24 overexpression resulted in the upregulation of PTEN as well as a reduction of Akt phosphorylation in HCT116 cells, but had no effect on the expression of Akt (Figure 4D); meanwhile, MMP‐2 and MMP‐9 expression was significantly downregulated in CRC cells compared to the control. In contrast, RBM24 knockdown produced the opposite effect (Figure 4E).

Further, the decreased expression of MMP‐2, MMP‐9 and phosphorylated Akt in CRC cells with RBM24 overexpressing could be reversed by exposure to the PTEN specific inhibitor SF1670 (Figure 4F). Additionally, RBM24 knockdown contributed to the significant downregulation of PTEN expression, increase in MMP‐2 and MMP‐9 expression and Akt phosphorylation compared to the control. However, Akt‐specific inhibitor MK‐2206 or PI3K inhibitor PI3K‐IN‐6 treatment could reverse the results induced by RBM24 knockdown in HCT116 cells (Figure 4G, H). Together, these results indicate that RBM24 attenuates the PI3K/Akt signalling pathway by regulating PTEN expression, causing the suppression of CRC cell proliferation, migration and invasion.

### 3.4 PTEN inhibitor SF1670 abolishes the inhibitory effect of RBM24 overexpression on CRC cells

Given our results showing that RBM24 expression was positively associated with PTEN expression and negatively influenced the PI3K/Akt signalling pathway, CRC cells overexpressing RBM24 were subjected to treatment with PTEN inhibitor SF1670. As expected, overexpression of RBM24 in HCT116 cells followed by treatment with 1 μM of SF1670 reversed the weakened cell proliferative, migratory and invasive capabilities induced by RBM24 overexpression (Figure 4I–K). Western blotting and qPCR analysis demonstrated that RBM24 overexpression promoted P21 and E‐cadherin expression, but attenuated the expression of CDK4, CyclinD1, MMP2/9 and Vimentin. However, the expression levels of the above‐mentioned genes were reversed by PTEN inhibitor SF1670 (Figure 4L, M).

In addition, RBM24 knockdown in combination with SF1670 treatment had a synergistic effect in promoting CRC cell migration and invasion, in upregulating MMP2/9 and Vimentin expression and in reducing E‐cadherin expression compared to the untreated cells (Figure S2A–F). Besides, proliferative, migratory and invasive capabilities of RBM24‐overexpressing HCT116 cells were restored by downregulation of PTEN expression using siRNA, whereas upregulation of PTEN expression by transfecting *pcDNA3.1*‐PTEN in RBM24‐knockdown HCT116 cells suppressed the malignant phenotypes of RBM24‐knockdown HCT116 cells (Figure S2G–J).

To determine the effect of RBM24 on PI3K/Akt signalling pathway, PI3K inhibitor PI3K‐IN‐6, or Akt inhibitor MK‐2206 was used. We found that PI3K‐IN‐6 or MK‐2206 treatment further suppressed the proliferation, migration and invasion of RBM24‐overexpressing CRC cells; meanwhile, a synergistic effect of RBM24 overexpression in combination with either PI3K‐IN‐6 or MK‐2206 treatment on gene expression was observed in comparison with untreated controls (Figure S3).

Conversely, RBM24 knockdown facilitated the malignant behaviour of HCT116 cells compared to the control; however, PI3K‐IN‐6 or MK‐2206 treatment could partially reverse the phenotypes induced by RBM24 knockdown. Meanwhile, PI3K‐IN‐6 or MK‐2206 treatment restored the expression of the above‐mentioned genes in RBM24‐knockdown HCT116 cells (Figure S4). Naturally, treatment with SF1670, PI3K‐IN‐6 or MK‐2206 alone influenced the proliferative, migratory and invasive capacities of CRC cells, respectively (Figure S5A–F). These findings suggest that RBM24 exerts its biological function by controlling PTEN expression and the PI3K/Akt signalling pathway.

### 3.5 RBM24 overexpression promotes chemotherapeutic agent‐induced apoptosis of CRC cells

**FIGURE 5:**
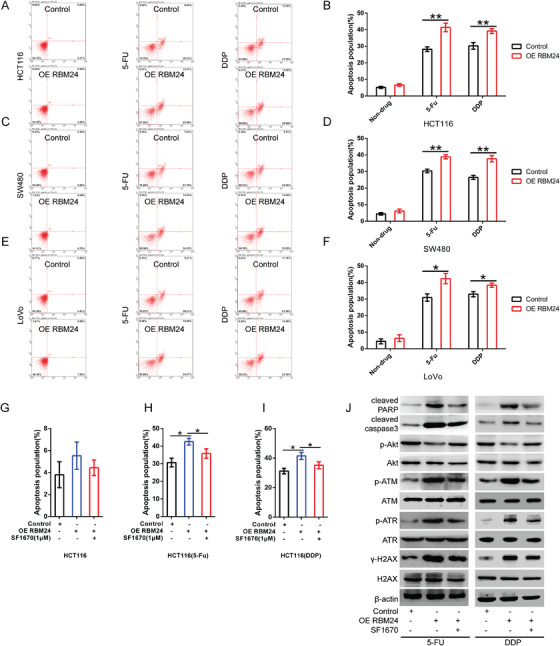
RBM24 promotes chemotherapeutic drug‐induced apoptosis of CRC cells. (A, B) The apoptosis rate of RBM24‐overexpressing HCT116 cells that were untreated or treated with 2 μg/ml of 5‐FU or 1 μg/ml of DDP. (B) Quantification. (C, D) The apoptosis rate of RBM24‐overexpressing SW480 cells untreated, or treated with 2 μg/ml of 5‐FU or 1 μg/ml of DDP. (D) Statistical analysis. (E, F) The apoptosis rate of RBM24‐overexpressing LoVo cells that were untreated or treated with 2 μg/ml of 5‐FU or 1 μg/ml of DDP. (F) Quantification. (G) The apoptosis rate of RBM24‐overexpressing HCT116 cells following treatment with 1 μM of SF1670 for 48 h. (H, I) SF1670 treatment (1 μM) for 48 h decreased the sensitivity of RBM24‐overexpressing HCT116 cells to 5‐FU or DDP, as compared with the cells treated with 5‐FU or DDP for 48 h alone. (J) The protein levels of cleaved Caspase3 and cleaved PARP, the phosphorylation levels of Akt, ATR, ATM and H2AX were analysed by Western blotting analysis in RBM24‐overexpressing HCT116 cells that were co‐treated with SF1670 and 5‐FU/DDP for 48 h. Data were analysed by two‐tailed Student's *t*‐test. Multiple comparisons were performed using one‐way ANOVA with post hoc LSD test. Error bars represented as S.D from at least three independent experiments. *p<.05, **p<.01. 5‐FU, 5‐fluorouracil; DDP, cis‐diamminedichloroplatinum

Our findings confirmed that overexpression or knockdown of RBM24 alone had no impact on the apoptosis of CRC cells compared with the controls. Nevertheless, RBM24 overexpression increased the apoptosis of CRC cells treated with fluorouracil (5‐FU) or cisplatin (Figure 5A–F) compared to the cells treated with 5‐FU or cisplatin alone. Besides, PTEN inhibitor SF1670 treatment reduced, but PI3K‐IN‐6 or MK‐2206 treatment increased 5‐FU or cisplatin‐induced apoptosis in RBM24‐overexpressing HCT116 cells (Figures 5G–I and S5G–I). On the contrary, RBM24 knockdown decreased 5‐FU or cisplatin‐induced apoptosis in CRC cells compared to the cells treated with 5‐FU or cisplatin alone (Figure S6A–F). PI3K‐IN‐6 or MK‐2206 treatment partially reversed, but SF1670 further elevated 5‐FU or cisplatin‐induced apoptosis in RBM24‐knockdown CRC cells as compared to the matched controls (Figure S6G–L).

ATM (ataxia‐telangiectasia, mutated) and ATR (ATM and Rad3‐related), belonging to protein kinases, are the sensors of DNA damage.^31^, ^32^ Gamma‐H2AX (γH2AX), a phosphorylated protein, is a marker of DNA double‐strand breaks.^33^, ^34^ In this study, western blotting analysis revealed that 5‐FU or cisplatin treatment significantly upregulated the expression of cleaved‐caspase3 and cleaved‐PARP (two markers of apoptotic induction) and the phosphorylation of ATM, ATR and H2AX in RBM24 overexpressing HCT116 cells as compared to the cells treated with 5‐FU or cisplatin alone; however, the above‐mentioned results could reverse by SF1670 treatment, or enhanced by MK‐2206. Conversely, as compared to HCT116 cells treated with 5‐FU or cisplatin alone, RBM24 knockdown significantly reduced the elevated phosphorylation levels of ATM, ATR and H2AX caused by 5‐FU or cisplatin, while MK‐2206 treatment restored the results induced by RBM24 knockdown (Figures 5J, S5J and S6M). Overall, these data suggest that RBM24 expression is associated with resistance of CRC cells to 5‐FU or cisplatin by regulating the PI3K/Akt signalling pathway. Considering the association among PI3K/Akt signalling pathway, tumour drug resistance and the regulation of PI3K/Akt signalling pathway by RBM24, we believe that RBM24 is closely related to CRC chemoresistance.

### 3.6 RBM24 directly binds to PTEN mRNA and enhances its mRNA stability

**FIGURE 6:**
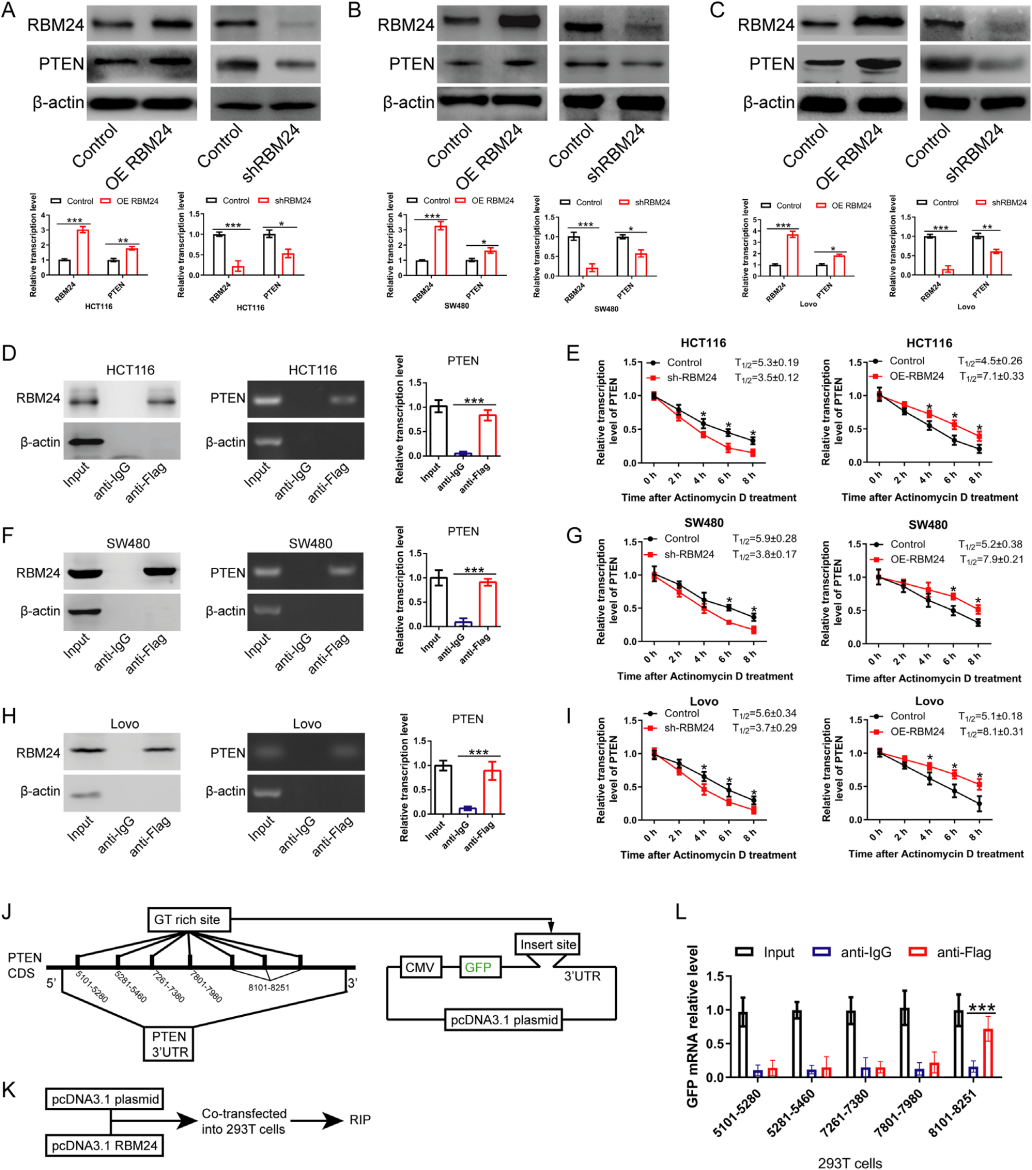
RBM24 directly binds PTEN mRNA and enhances its mRNA stability. (A–C) The mRNA and protein levels of PTEN and RBM24 in RBM24‐overexpressing/knockdown (A) HCT116, (B) SW480 and (C) LoVo CRC cells. (D) The interaction between RBM24 and PTEN in HCT116 cells was assessed by RIP assay, Western blotting and qPCR analysis. (E) After treatment of HCT116 cells with 5 μg/ml of actinomycin D, the effects of RBM24 knockdown (left) or overexpression (right) on PTEN mRNA stability at different time points were measured by qPCR analysis. (F) The interaction between RBM24 and PTEN in SW480 cells was validated by RIP assay, Western blot and qPCR analysis. (G) After treatment of SW480 cells with 5 μg/ml actinomycin D, the effects of RBM24 knockdown (left) or overexpression (right) on PTEN mRNA stability at different time points were monitored by qPCR analysis. (H) The interaction between RBM24 and PTEN levels in LoVo cells was evaluated by RIP assay, Western blot and qPCR analysis. (I) After treatment of LoVo cells with 5 μg/ml actinomycin D, the effect of RBM24 knockdown (left) or overexpression (right) on PTEN mRNA stability at different time points were detected by qPCR analysis. (J–K) Schematic diagrams of experimental design. (L) RIP experiment was performed to detect the interaction between RBM24 and the GT‐rich sites of PTEN mRNA. Data were analysed by two‐tailed Student's *t*‐test or one‐way ANOVA with post hoc LSD test. Error bars represented as SD from at least three independent experiments. *p<.05, **p<.01, ***p<.001

To understand the specific mechanism that RBM24 regulates PTEN expression in CRC, RBM24 was overexpressed or silenced in various types of CRC cells (HCT116, SW480, LoVo). Our findings revealed that expression levels of PTEN were correlated with that of RBM24 (Figure 6A–C). To determine whether RBM24 directly binds to PTEN mRNA, RNA immunoprecipitation (RIP) assay was performed. Data showed that PTEN mRNA co‐precipitated with RBM24, indicating a direct interaction between RBM24 protein and PTEN mRNA (Figure 6D, F, H). To further evaluate the effect of RBM24 on the stability of PTEN mRNA, CRC cells were treated with the transcriptional inhibitor actinomycin D and mRNA decay was assessed by qPCR analysis. RBM24 knockdown resulted in a decrease of the half‐life of PTEN mRNA, whereas overexpression of RBM24 extended its half‐life time (Figure 6E, G, I), suggesting that RBM24 stabilised PTEN mRNA. These data demonstrate that RBM24 promotes PTEN mRNA stability by directly binding to its 3′‐UTR regions.

Considering that previous studies have proved that the RNA binding site of RBM24 is a GT‐rich region, we further cloned the GT‐rich region in the 3′‐UTR region of PTEN mRNA into plasmid *pcDNA3.1* expressing GFP (Figure 6J). Thereafter, *pcDNA3.1‐PTEN 3′‐UTR* and *pcDNA3.1‐RBM24* were co‐transfected into 293T cells (Figure 6K). Results from RIP and qPCR assays showed that among the 5 GT‐rich regions in the 3′‐UTR of PTEN mRNA, only the GT‐rich regions at positions 8101–8251 were able to pull down GFP mRNA from total RNA (Figure 6L). Therefore, these findings suggest that RBM24 enhances the stability of PTEN mRNA by directly binding to the GT‐rich region at positions 8101–8251 in the 3′‐UTR of PTEN mRNA.

### 3.7 Rbm24 is downregulated in colorectal adenoma tissues of Apc^min/+^ mice

**FIGURE 7:**
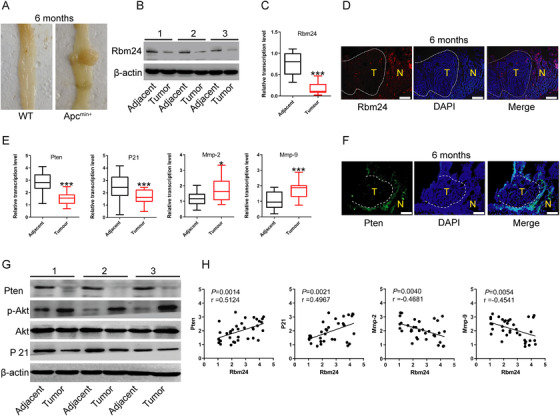
Rbm24 is downregulated in colorectal adenoma tissues of Apc^min/+^ mice. (A) Representative images of intestinal tissues from 6‐month‐old WT and Apc^min/+^ mice. (B) Western blot, (C) qPCR and (D) immunofluorescence assays were performed to detect the expression of Rbm24 in the para‐cancerous normal and intestinal adenoma tissues from Apc^min/+^ mice (*N* = 18 mice). Scale bar = 200 μm. (E) The mRNA levels of Pten, P21, Mmp‐2 and Mmp‐9 in intestinal adenoma or adjacent normal tissues of Apc^min/+^ mice. (F) PTEN protein expression in intestinal tumour tissues of Apc^min/+^ mice was detected by immunofluorescence. Scale bar = 100 μm. (G) Western blotting analysis was carried out to evaluate the protein levels of Pten, P21, total Akt and phosphorylated Akt in adjacent normal tissues and intestinal adenomas of Apc^min/+^ mice. (H) The correlation between the mRNA expression levels of Rbm24 and Pten, or P21, or Mmp‐2, or Mmp‐9 in intestinal tumour tissues and adjacent normal tissues of Apc^min/+^ mice. Data were expressed as the mean ± SD from at least three independent experiments and analyzed using the two‐tailed Student's *t*‐test or Pearson's correlation test. *p<.05, ***p<.001. T, tumour tissues; N, adjacent normal tissues

APC suppresses CRC development by repressing the Wnt signalling pathway.^35^ APC gene mutation and inactivation are often observed in CRC. Apc^min/+^ mice spontaneously develop intestinal adenomas, which is the classic animal model for studying CRC (Figure 7A). In this study, we found that Rbm24, PTEN and P21 mRNA levels were significantly downregulated in tumour tissues of Apc^min/+^ mice compared to adjacent normal samples (*N* = 18); meanwhile, the expression levels of MMP‐2 and MMP‐9 were markedly elevated (Figure 7B–F). Notably, when the protein expression of PTEN was downregulated, Akt phosphorylation was significantly increased and total Akt was not changed in tumour tissues of Apc^min/+^ mice, compared with para‐cancer tissues (Figure 7G). Besides, the expression levels of MMP‐2, MMP‐9 and P21, the downstream genes of PI3K/Akt pathway, was also significantly altered, of which MMP‐2 and MMP‐9 expression were increased, but P21 expression was decreased. Further correlation analysis showed a statistically significant positive correlation between the transcriptional levels of PTEN or P21 and Rbm24, an inverse correlation between MMP‐2/MMP‐9 expression and Rbm24 expression (Figure 7H).

### 3.8 RBM24 is downregulated in human CRC tissue samples and low RBM24 expression is associated with poor prognosis in CRC patients

**FIGURE 8:**
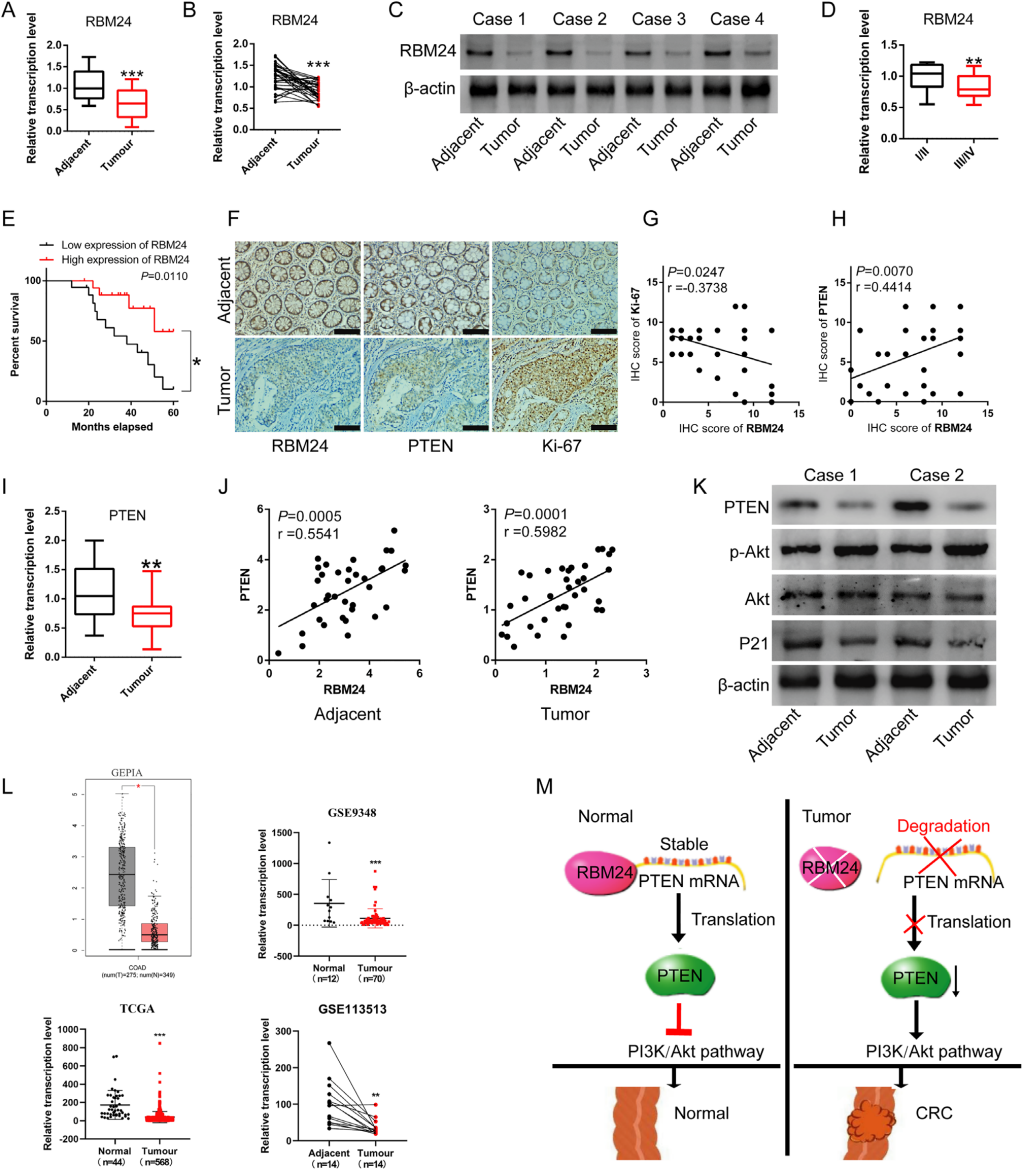
RBM24 is downregulated in human colorectal cancer and associated with prognosis in CRC patients. (A–C) RBM24 expression in human colorectal cancer tissues and paired normal tissues was tested by (A, B) qPCR and (C) Western blotting analysis (*N* = 36). (D) RBM24 expression in patients with stage III and IV Colorectal Cancer and in patients with stage I and II. (E) Low expression of RBM24 predicted a worse prognosis in patients with colorectal cancer. (F) Immunohistochemistry was performed to detect the protein levels of RBM24, Ki‐67 and PTEN in human colorectal cancer tissues and matched normal tissues. Scale bar = 100 μm. (G) Correlation between the RBM24 and Ki‐67 IHC score. (H) Correlation between the RBM24 and PTEN IHC score. (I, J) PTEN expression and its correlation with RBM24 level in human colorectal cancer tissues and paired normal samples. (K) The phosphorylated Akt, total Akt level, the protein levels of P21 and PTEN in human colorectal cancer tissues and paired normal tissues were tested by Western blotting analysis. (L) GEPIA, GEO and TCGA datasets showed RBM24 expression in human colorectal cancers. (M) Schematic representation of the role of RBM24 in colorectal tumourigenesis. Data were analyzed by two‐tailed Student's *t*‐test. Error bars represented as S.D from at least three independent experiments. *p<.05, **p<.01, ***p<.001

To further explore the role of RBM24 in human CRC, we examined RBM24 expression in human CRC tissues and paired para‐cancerous samples. qPCR and western blotting analysis revealed that, compared with adjacent normal tissues, RBM24 expression in human CRC tissues was significantly reduced (Figure 8A–C), an effect that was also associated with TNM staging (Figure 8D) and overall survival (*N* = 36; Figure 8E). There was no correlation between RBM24 and other clinical characteristics, including age and gender of CRC patients. Immunohistochemistry (IHC) indicated that RBM24 and PTEN expression in human CRC samples was significantly lower than in para‐cancerous tissues (Figures 8F and S7). Besides, Ki‐67, a proliferation marker, was significantly upregulated in CRC tissues compared to adjacent normal samples (Figures 8F and S8A). Correlation analysis showed a negative correlation between the IHC scores of RBM24 and Ki‐67 (Figure 8G) and a positive correlation between the IHC scores of RBM24 and PTEN (Figure 8H).

Further, qPCR results showed that mRNA expression of PTEN in human CRC tumour tissues was markedly downregulated (Figure 8I) and positively correlated with mRNA expression of RBM24 (Figure 8J). However, MMP‐2 and MMP‐9 expression in tumour tissues were significantly upregulated and both were negatively correlated with RBM24 expression; P21 expression was inversely correlated with MMP‐2/MMP‐9 expression (Figure S8B–G). Western blotting analysis confirmed that PTEN and P21 expression were downregulated in tumour tissues; Akt phosphorylation was significantly upregulated and no change was observed in the expression level of total Akt (Figure 8K).

Similarly, by searching the GEPIA (Gene Expression Profiling Interactive Analysis) (http://gepia.cancer‐pku.cn/), GEO and TCGA datasets, we found that RBM24 was significantly downregulated in CRC tumour tissues (Figure 8L). PTEN expression was positively correlated with RBM24 expression in CRC (Figure S8H). Notably, ROC curves showed that RBM24 level could distinguish tumours from normal tissues (Figure S8I). RBM24 expression level in colon adenocarcinoma (COAD) and rectum adenocarcinoma (READ) was inversely correlated with its promoter methylation level (Figure S8J). Besides, there was no relationship between RBM24 expression and APC or KRAS mutation in CRC (Figure S8K), indicating that the expression of RBM24 may be epigenetically affected in tumours by aberrant methylation.

Overall, schematic diagram of how RBM24 exerts it function in CRC tumourigenesis is represented (Figure 8 M). Briefly, RBM24 directly binds to the 3′‐UTR of PTEN mRNA and maintains its mRNA stability, leading to the increased PTEN protein levels, thereby repressing PI3K/Akt signalling pathway. Nevertheless, abnormally low expression of RBM24 promotes degradation of PTEN mRNA, resulting in decreased PTEN protein expression, thereby relieving its inhibitory effects on PI3K/Akt pathway. Taken together, our findings suggest that RBM24 acts as a tumour suppressor gene in colorectal tumourigenesis.

## 4 DISCUSSION

Although a previous study has reported that RBM24 presents a certain correlation with Hirschsprung's disease,^36^ little is known about the biological function of RBM24 in intestinal tumours. In the present study, our findings demonstrate that RBM24 plays a pivotal role in controlling CRC tumourigenesis. To fully elucidate the role of RBM24 in the development of colorectal adenoma or CRC, an Rbm24 conditional knockout mouse model was successfully constructed. Data indicated that Rbm24 knockout promoted the proliferation of colorectal epithelial cells in mice, increased intestinal length and facilitated the development of colorectal adenomas in mice. In addition, RBM24 is involved in the regulation of the cell cycle in a variety of terminally differentiated cells,^21^, ^22^, ^23^ indicating that RBM24‐mediated cell cycle progression widely exists in living cells. Here, CRC cells overexpressing or silencing RBM24 were established. Via cell proliferation assays, colony formation assay and transwell assays, we confirmed that RBM24 was closely associated with CRC cell proliferation, migration and invasion. Moreover, RBM24 was associated with the sensitivity of CRC cells to 5‐FU and to cisplatin. In human CRC tissues, RBM24 is significantly downregulated compared to matched normal tissues. As CRC patients with higher expression of RBM24 live longer, we hypothesise that RBM24 has a certain correlation with the recurrence of CRC or drug resistance. Mechanistically, RBM24 exerts its biological functions by directly binding to the mRNA of PTEN. The downregulated expression of RBM24 leads to lower expression of PTEN. Subsequently, the decreased expression of PTEN protein relieves the inhibitory effect on PI3K/Akt signalling pathway. Subsequently, activation of the PI3K/Akt signalling axis promotes the malignant phenotype of CRC cells. Taken together, these results show that RBM24 acts as a tumour suppressor gene, suppressing colorectal tumourigenesis.

Previous studies have shown that RBM24 also participates in cytoskeletal organisation,^20^ providing new insight into the biological function of RBM24 in CRC. Cytoskeletal regulation is significantly associated with cell migration and motor ability.^37^, ^38^ Therefore, we assumed that RBM24 knockout could increase the migratory capacity of cells and favour cell migration, which is also one of the hallmark events of CRC development.^12^ After overexpressing or silencing RBM24 expression, we found that the cell migratory and invasive abilities and the expression level of EMT‐related genes (E‐cadherin and vimentin) were significantly changed, that is, RBM24 expression negatively regulated CRC cell migration and invasion.

Apc^min/+^ mouse model is a classic animal model used in CRC research. The mutation of the APC gene contributes to the abnormal activation of the Wnt/β‐catenin signalling pathway, which results in interference with the expression of multiple genes, initiating colorectal adenoma in Apc^min/+^ mice.^39^, ^40^, ^41^ In this study, our data indicated that Rbm24 was significantly downregulated in intestinal tumour of Apc^min/+^ mice. Nevertheless, we did not find an association between RBM24 expression and APC mutation in human colorectal cancer tissues via bioinformatics analysis. This may be due to differences in the regulation of gene expression between human and murine. In addition, RBM24 expression in tumour tissues of CRC patients was dramatically reduced and the expression of RBM24 correlated significantly with the prognosis of CRC patients, which was consistent with the data from TCGA and GEPIA databases. CRC patients with high expression of RBM24 seemed to have better long‐term survival. Therefore, we considered that RBM24 may be related to drug resistance of CRC, which was confirmed to a certain extent in this study. High expression of RBM24 elevated 5‐FU or cisplatin‐induced apoptosis rate. However, since these results were obtained only in vitro, the mechanisms by which the downregulation of RBM24 lessens sensitivity of CRC cells to chemotherapy agents need to be further explored.

Previous studies have indicated that RBM24 can bind several tumour‐related genes.^21^, ^22^, ^24^ PTEN, a phosphatase on PIP3, acts as a tumour suppressor gene and activates PI3K/Akt signalling pathway.^42^, ^43^ Moreover, the downstream target genes of Akt, including CDK4, MMP‐2 and MMP‐9, are involved in cancer cell proliferation, migration and invasion. RBM24 is highly homologous to RBM38^44^; the biological functions of RBM24 and RBM38 share some similarities. In a previous study, it was reported that RBM38 functions as a tumour suppressor, which can bind PTEN mRNA and maintain its stability, increasing mRNA levels of PTEN in breast cancer tissue.^45^ In the present study, our results suggested that RBM24 could bind to the mRNA of PTEN and maintain its stability, thereby regulating the PI3K/Akt signalling pathway. This notion was supported by evidence that treatment with PI3K/Akt signalling inhibitors could partially reverse phenotypes induced by RBM24 knockdown. P21 is regulated by RBM24,^24^ which represses the proliferation of various tumour cells.^46^ However, it has also been shown that P21 is a downstream molecule of the PI3K/Akt pathway.^47^ Accordingly, our results indicate that overexpression of RBM24 in CRC cells followed by treatment with PTEN inhibitor could partially reverse phenotypes induced by RBM24 overexpression. In the RIP assay, our results further confirmed that RBM24 directly binds to PTEN mRNA, which strongly suggests that RBM24 affects PTEN mRNA abundancy and leads to the activation of its downstream signalling pathways.

## 5 CONCLUSIONS

In conclusion, our findings revealed that RBM24 suppressed colorectal tumourigenesis by increasing the stability of PTEN mRNA, which led to the suspension of the PI3K/Akt signalling axis. Since the RBM24‐mediated regulatory pathway represses CRC development, targeting RBM24 holds strong promise for the diagnosis and treatment of CRC. To our knowledge, this is the first study to show that RBM24 plays an important tumour suppressive role in colorectal tumourigenesis in an animal model, which is invaluable for understanding the biological function of RBM24 in cancer.

## AUTHORS’ CONTRIBUTIONS

RMX and TL: collection and/or assembly of data, data analysis, interpretation and manuscript writing; WGL: reviewed and approved the final manuscript; XQX: conception and design, financial support, administrative support, manuscript writing and final approval of manuscript. All authors read and approved the final manuscript.

## CONFLICT OF INTEREST

The authors declare that there is no conflict of interest.

## ETHICS APPROVAL AND CONSENT TO PARTICIPATE

Ethical approval was obtained from Xiamen University (approval no. XDYX2021008) and written informed consent was obtained from each patient.

## CONSENT FOR PUBLICATION

The authors agree to the publication of all the data involved in this article. No data from other entities are used in this study.

## Supporting information



Supporting Information

## Data Availability

The analysed data sets generated during the study are available from the corresponding author on reasonable request.
