# Supplementary material for: RNA‐binding protein RBM24 represses colorectal tumourigenesis by stabilising PTEN mRNA
Source: Clin Transl Med. 2021 Oct 12;11(10):e383. doi: 10.1002/ctm2.383 (PMC8506628; doi:10.1002/ctm2.383)

Supplementary Figure S1

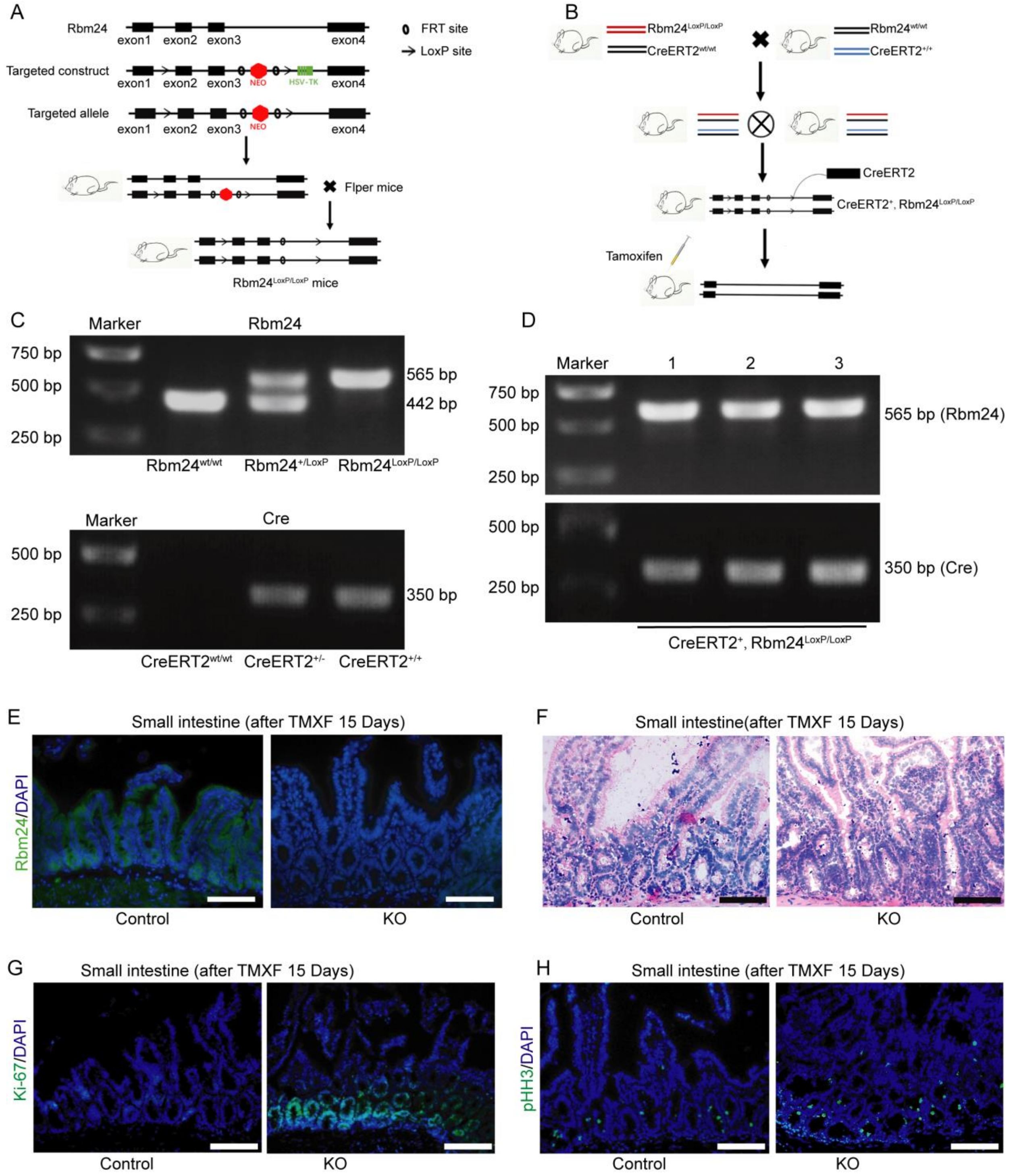

Supplementary Figure S2

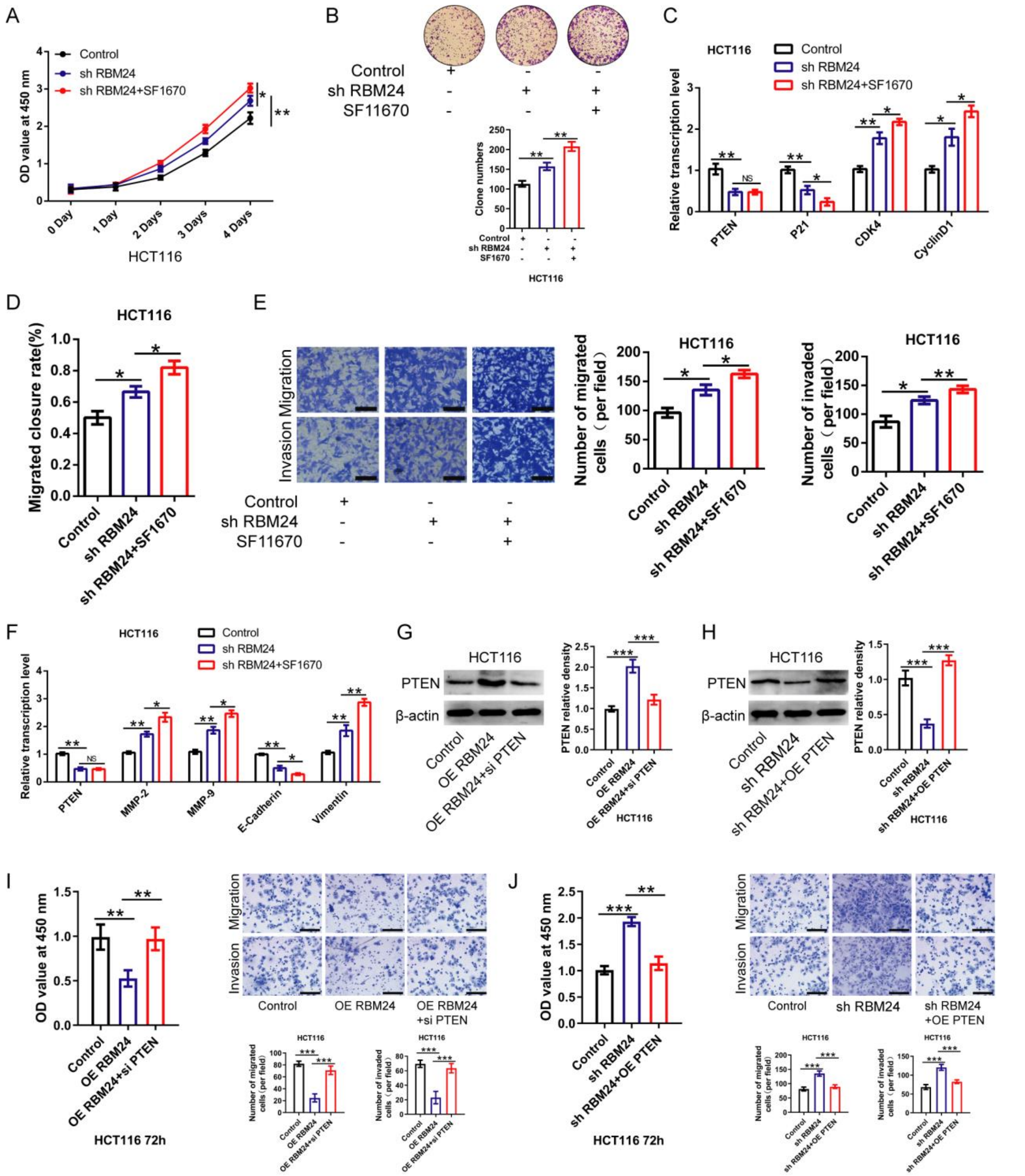

Supplementary Figure S3

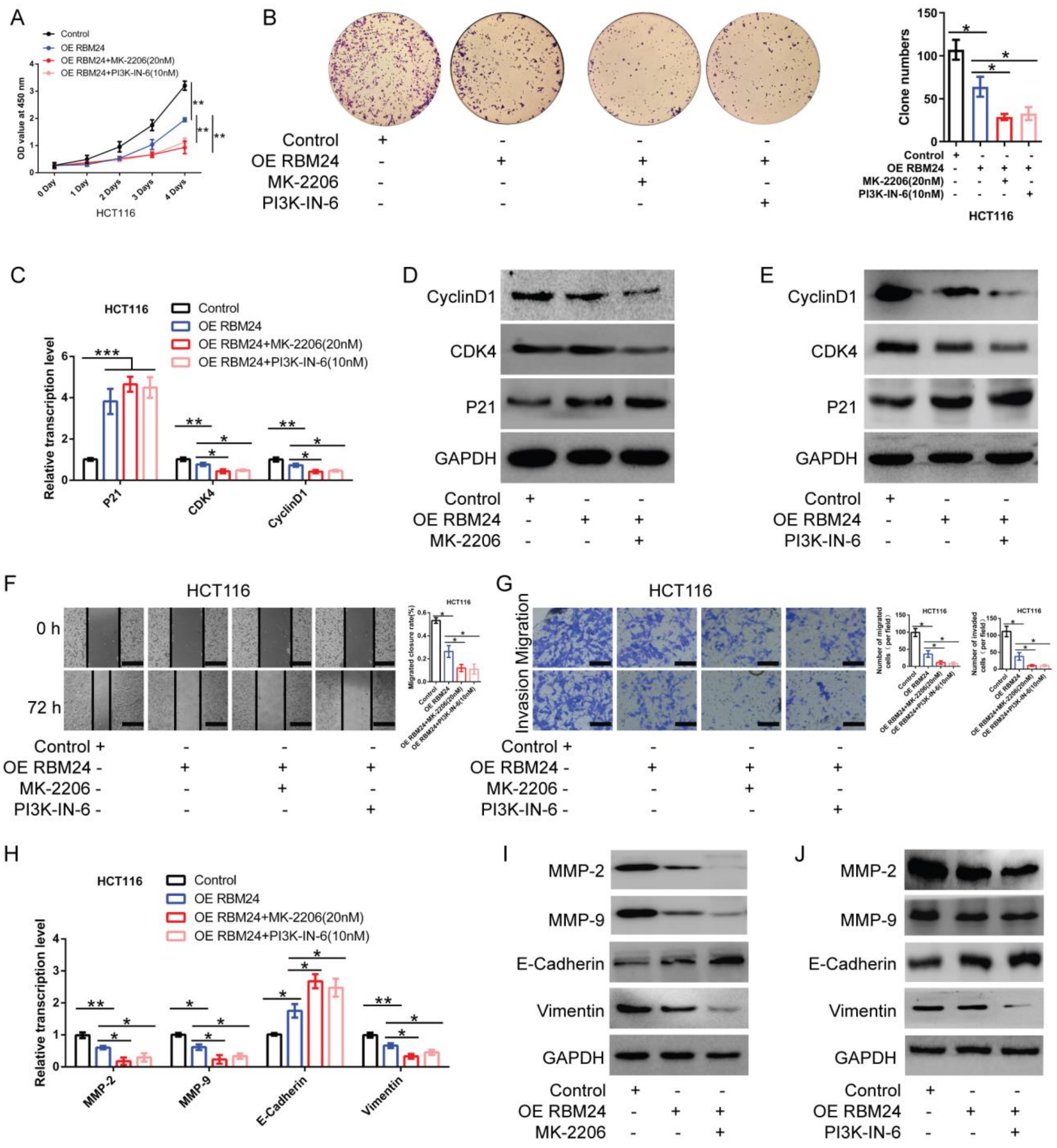

Supplementary Figure S4

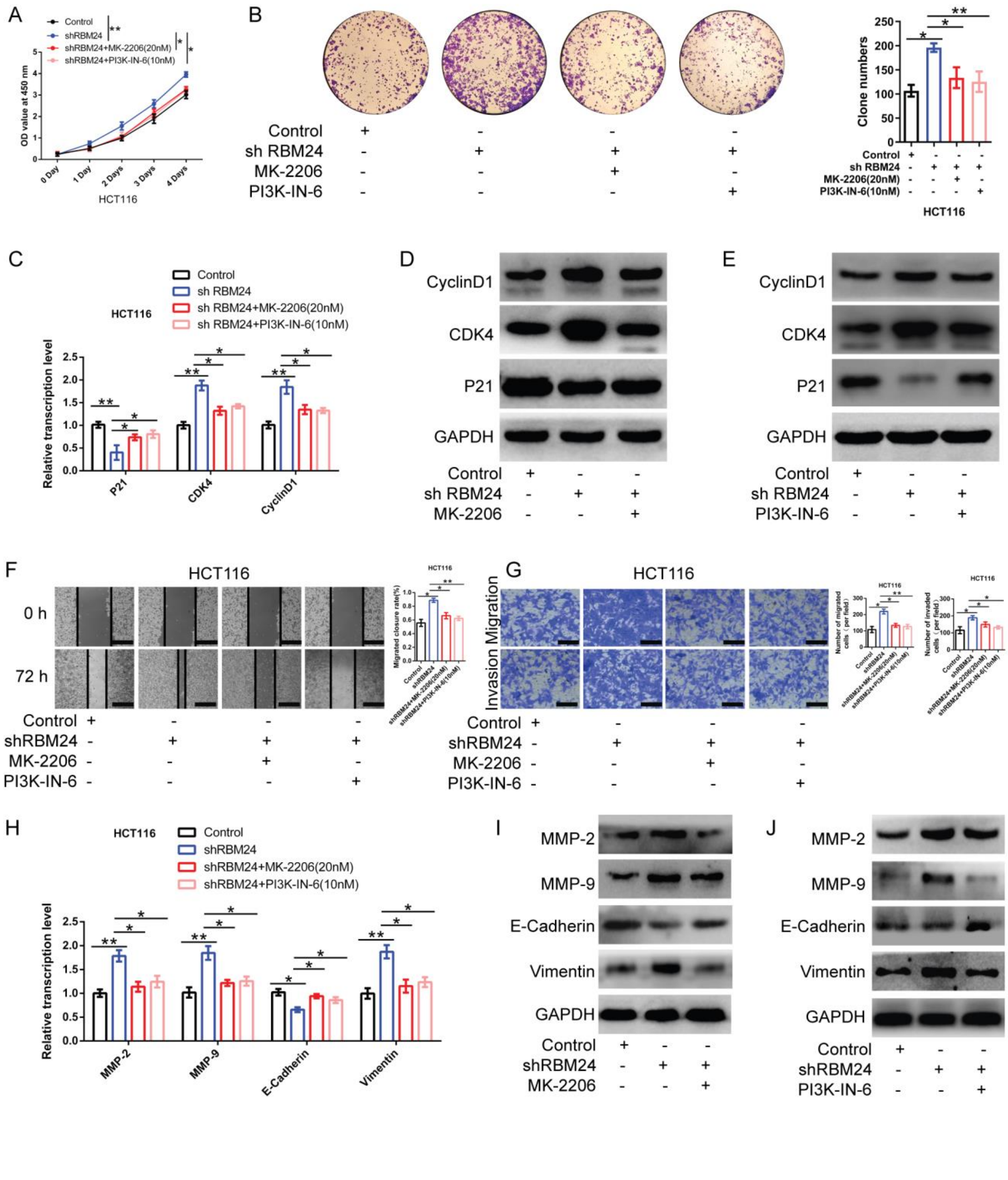

Supplementary Figure S5

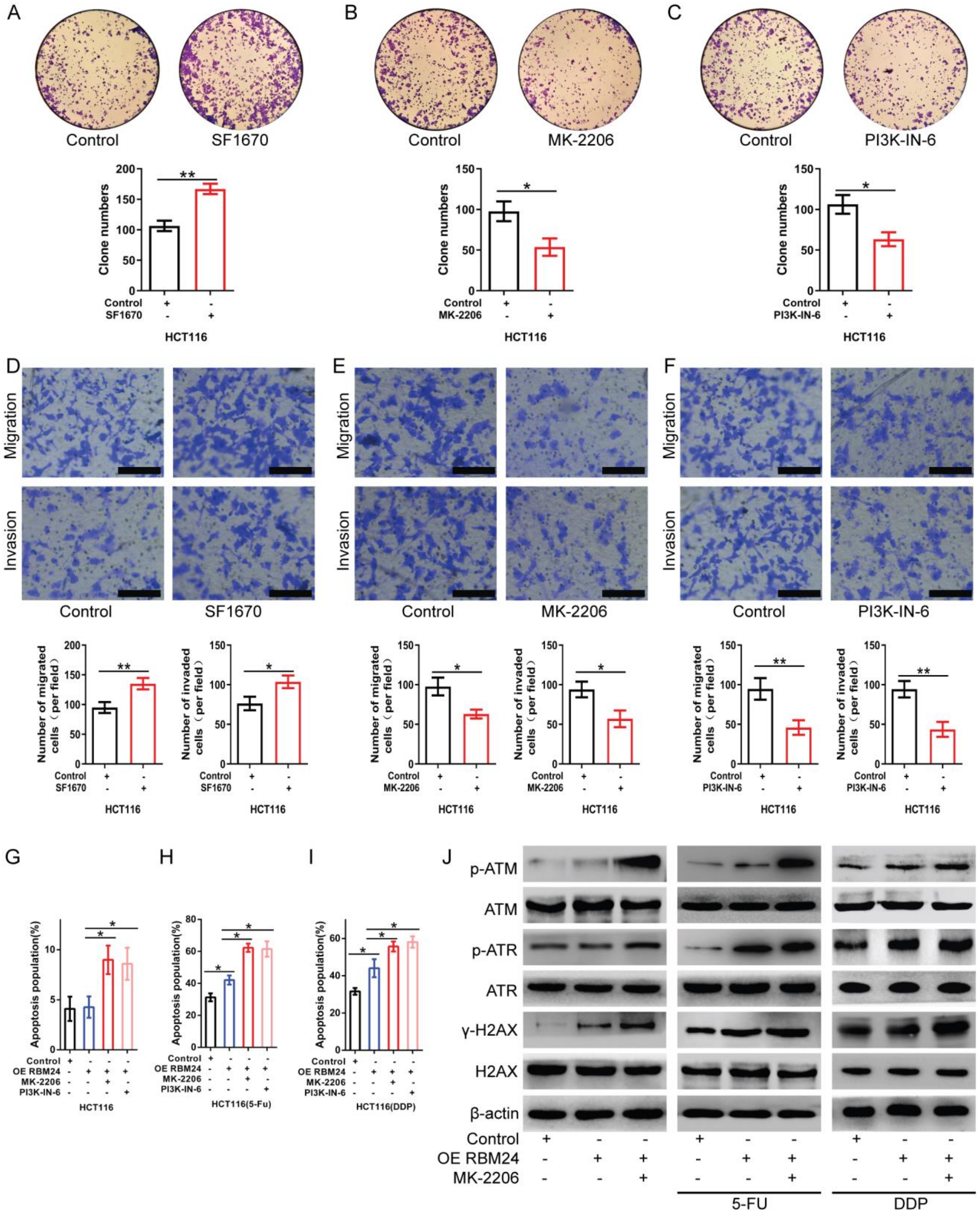

Supplementary Figure S6

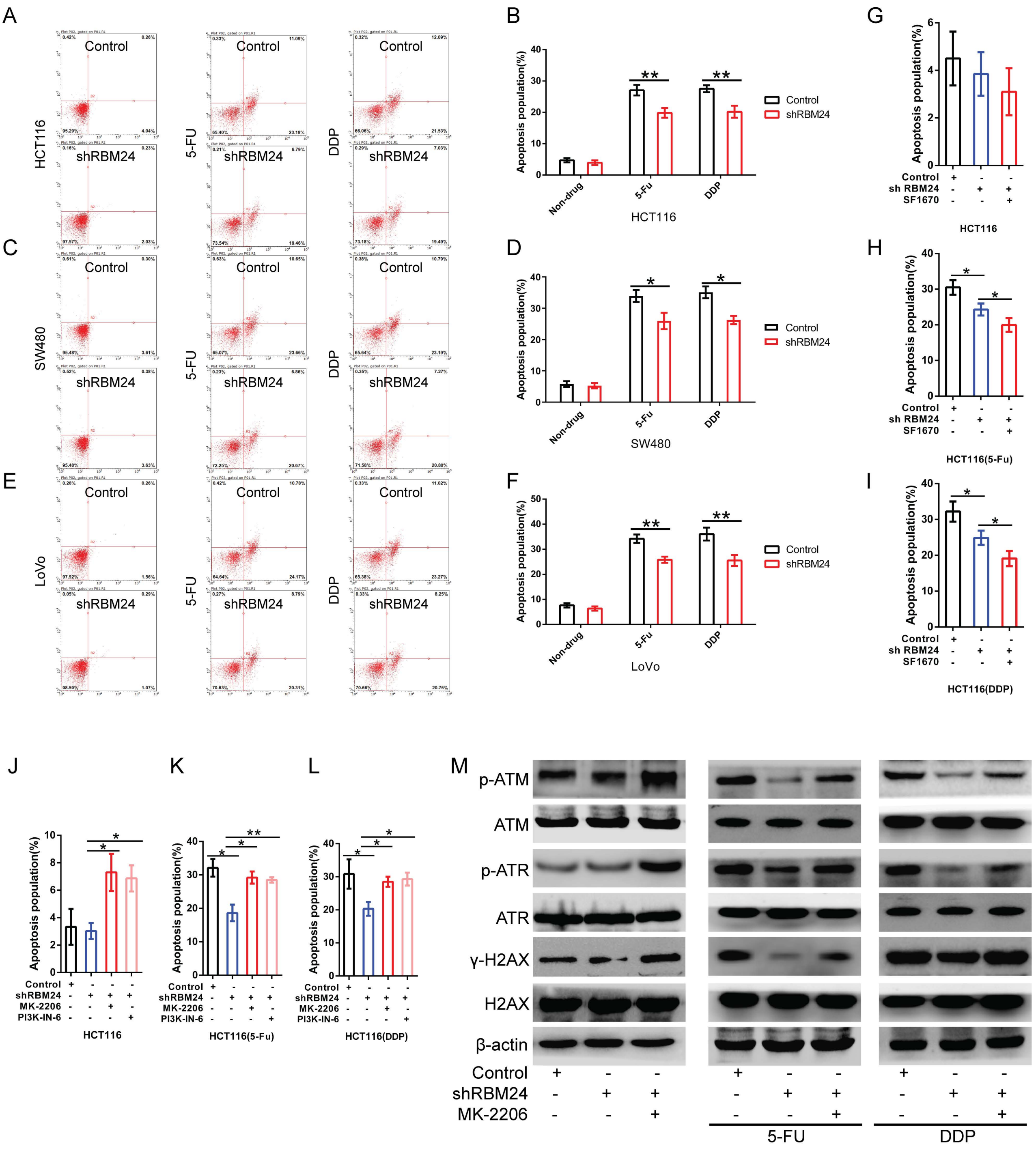

Supplementary Figure S7

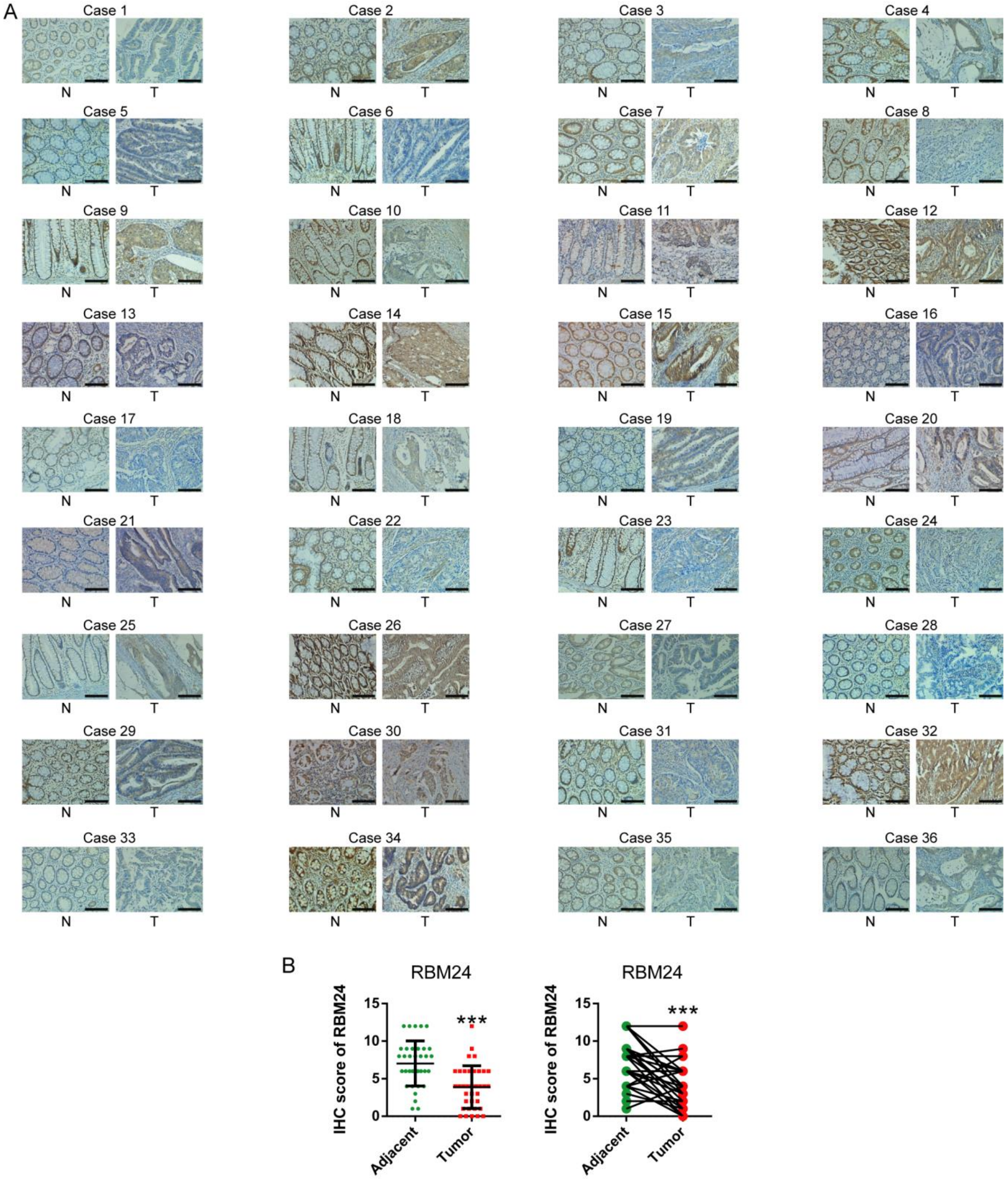

Supplementary Figure S8

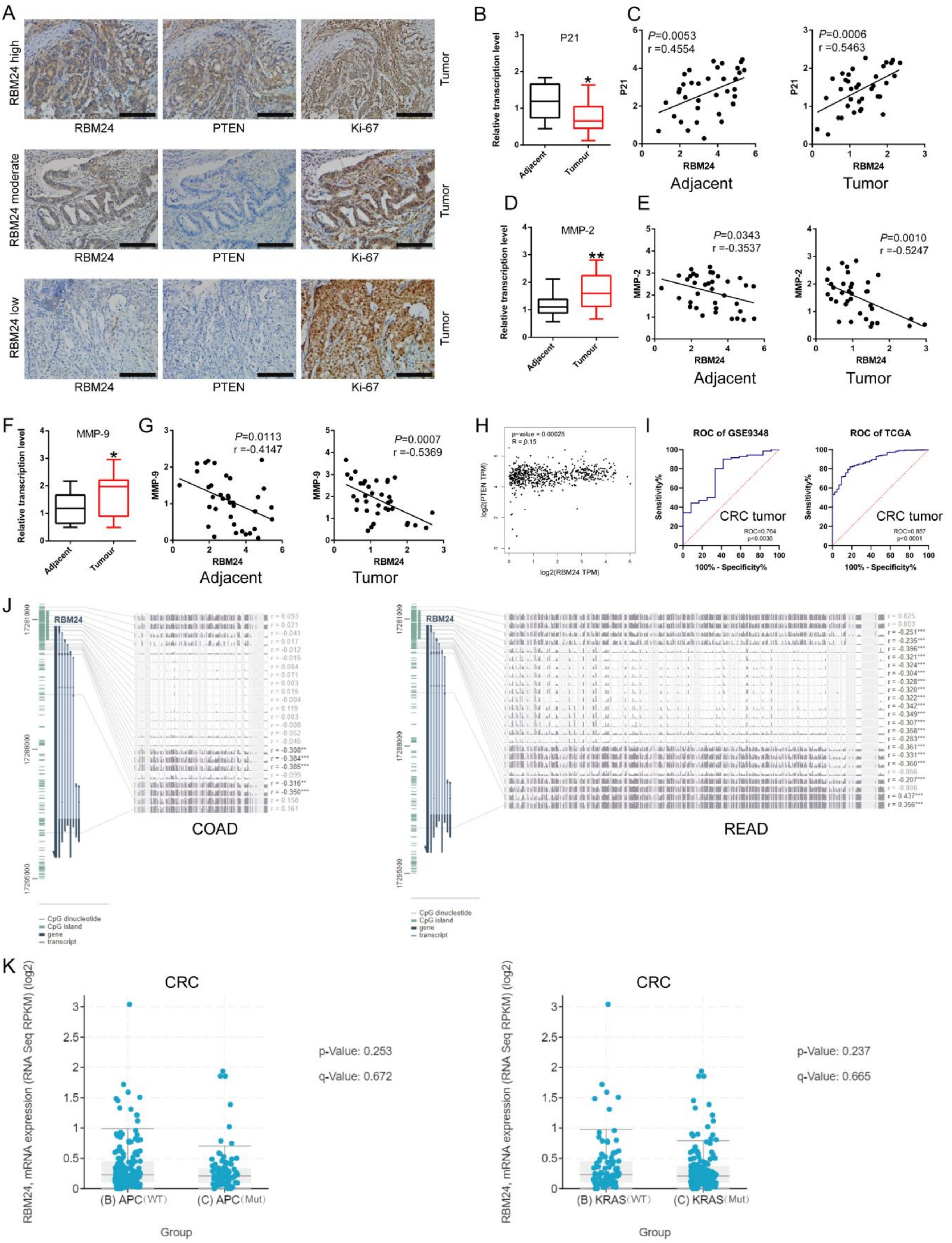

Supplement: Supplementary file 1 — Supporting Information [file CTM2-11-e383-s001.pdf]
